# Cross-species efficacy of enzyme replacement therapy for CLN1 disease in mice and sheep

**DOI:** 10.1172/JCI163107

**Published:** 2022-10-17

**Authors:** Hemanth R. Nelvagal, Samantha L. Eaton, Sophie H. Wang, Elizabeth M. Eultgen, Keigo Takahashi, Steven Q. Le, Rachel Nesbitt, Joshua T. Dearborn, Nicholas Siano, Ana C. Puhl, Patricia I. Dickson, Gerard Thompson, Fraser Murdoch, Paul M. Brennan, Mark Gray, Stephen N. Greenhalgh, Peter Tennant, Rachael Gregson, Eddie Clutton, James Nixon, Chris Proudfoot, Stefano Guido, Simon G. Lillico, C. Bruce A. Whitelaw, Jui-Yun Lu, Sandra L. Hofmann, Sean Ekins, Mark S. Sands, Thomas M. Wishart, Jonathan D. Cooper

**Affiliations:** 1 Department of Pediatrics, Washington University in St. Louis, School of Medicine, St. Louis, Missouri, USA.; 2 The Roslin Institute and Royal (Dick) School of Veterinary Studies, University of Edinburgh, Easter Bush Campus, Easter Bush, Scotland, United Kingdom.; 3 Department of Medicine, Washington University in St. Louis, School of Medicine, St .Louis, Missouri, USA.; 4 Discovery Science Division, Amicus Therapeutics Inc., Philadelphia, Pennsylvania, USA.; 5 Collaborations Pharmaceuticals Inc., Lab 3510, Raleigh, North Carolina, USA.; 6 Department of Genetics, Washington University in St. Louis, School of Medicine, St. Louis, Missouri, USA.; 7 Centre for Clinical Brain Sciences, University of Edinburgh, Chancellor’s Building, Edinburgh, Scotland, United Kingdom.; 8 Department of Clinical Neurosciences, NHS Lothian, Edinburgh, Scotland, United Kingdom.; 9 The Large Animal Research and Imaging Facility (LARIF), Royal (Dick) School of Veterinary Studies, University of Edinburgh, Easter Bush Campus, Easter Bush, Scotland, United Kingdom.; 10 Department of Internal Medicine, University of Texas Southwestern Medical Center, Dallas, Texas, USA.; 11 Department of Neurology, Washington University in St. Louis, School of Medicine, St. Louis, Missouri, USA.

**Keywords:** Neuroscience, Therapeutics, Lysosomes, Monogenic diseases, Neurodegeneration

## Abstract

CLN1 disease, also called infantile neuronal ceroid lipofuscinosis (NCL) or infantile Batten disease, is a fatal neurodegenerative lysosomal storage disorder resulting from mutations in the *CLN1* gene encoding the soluble lysosomal enzyme palmitoyl-protein thioesterase 1 (PPT1). Therapies for CLN1 disease have proven challenging because of the aggressive disease course and the need to treat widespread areas of the brain and spinal cord. Indeed, gene therapy has proven less effective for CLN1 disease than for other similar lysosomal enzyme deficiencies. We therefore tested the efficacy of enzyme replacement therapy (ERT) by administering monthly infusions of recombinant human PPT1 (rhPPT1) to PPT1-deficient mice (*Cln1^–/–^*) and *CLN1^R151X^* sheep to assess how to potentially scale up for translation. In *Cln1^–/–^* mice, intracerebrovascular (i.c.v.) rhPPT1 delivery was the most effective route of administration, resulting in therapeutically relevant CNS levels of PPT1 activity. rhPPT1-treated mice had improved motor function, reduced disease-associated pathology, and diminished neuronal loss. In *CLN1^R151X^* sheep, i.c.v. infusions resulted in widespread rhPPT1 distribution and positive treatment effects measured by quantitative structural MRI and neuropathology. This study demonstrates the feasibility and therapeutic efficacy of i.c.v. rhPPT1 ERT. These findings represent a key step toward clinical testing of ERT in children with CLN1 disease and highlight the importance of a cross-species approach to developing a successful treatment strategy.

## Graphical abstract

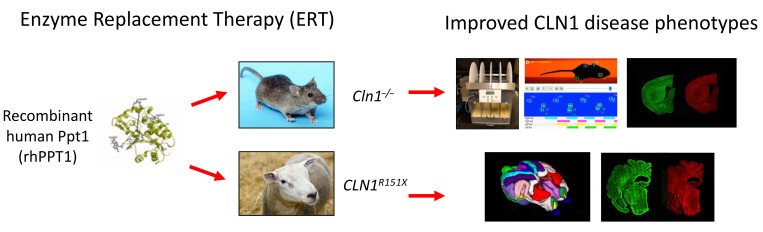

## Introduction

In contrast to other monogenic causes of neurodegeneration such as mitochondrial and peroxisomal disease (1, 2), the majority of fatal lysosomal storage disorders (LSDs) are caused by a deficiency in proteins that are amenable to exogenous supply (3). These are soluble lysosomal enzymes that can be secreted and taken up by cells via mannose-6-phosphate receptors (MPRs) in a process named “cross-correction” (4). Nonetheless, the blood-brain barrier prevents systemically delivered enzyme replacement therapy (ERT) from reaching the CNS, unless its uptake properties are modified (5). Intracerebrovascular (i.c.v.) delivery of recombinant enzyme is therefore an attractive option to overcome this problem and deliver ERT directly to the brain. However, across more than 40 neuronopathic LSDs, only 1 has an approved treatment available (6), so CNS-directed ERT still lacks general principles and procedures. Obstacles to developing ERT include manufacturing appropriately glycosylated and phosphorylated recombinant enzymes, defining appropriate dosing regimens, and delivering enzymes to reach all affected CNS regions (3). Importantly, there is a particular unmet need to scale up preclinical advances made in mouse models to large animal models to maximize the chances of successful clinical translation.

CLN1 disease, also called infantile neuronal ceroid lipofuscinosis (NCL) or infantile Batten disease, is a rapidly progressing, devastating neurodegenerative LSD (7). The classic form of CLN1 disease begins in infancy and can present as early as 6 months of age. However, some mutations in this gene result in more delayed presentations according to the precise mutation (8, 9). Its clinical manifestations include sensory and motor deficits, visual impairment leading to blindness, and epileptic seizures (10). Because there is currently no effective therapy, all cases are fatal (11, 12), with a life expectancy of 9 to 12 years (10). This disease results from mutations in the *CLN1* gene that encodes the soluble lysosomal enzyme palmitoyl protein thioesterase 1 (PPT1) (9, 13). Although CLN1 disease is theoretically amenable to an ERT strategy, developing this approach represents a greater challenge than for other LSDs. This is due to its early onset and aggressive disease course and the need to treat widespread regions of the CNS including both the brain and spinal cord (14, 15).

We have previously shown that administration of a single intrathecal (i.t.) or intravenous (i.v.) dose of recombinant human PPT1 (rhPPT1) to neonatal PPT1-deficient mice (*Cln1^−/−^*) produced modest effects on behavior and pathology (16–18). Because exogenously supplied rhPPT1 has a finite half-life (16), we hypothesized that repeated dosing of the CNS would be required to provide a translatable therapy.

In this study, we tested this repeated dosing strategy first in *Cln1^−/−^* mice, a well-established and -characterized model recapitulating most aspects of classical CLN1 disease (14, 15, 19), using a previously defined dose and frequency based on the enzyme’s half-life (16). We then applied the same strategy to a recently generated sheep model of CLN1 disease (20) that carries the most common human disease–causing null mutation (*CLN1^R151X^*). In this strategy, we used infusion parameters similar to those used in other large animal models of LSDs (21, 22). This sheep model allowed the assessment of enzyme biodistribution and dosing in a species with a brain size and complexity that approximates that of the human brain.

We determined the therapeutic efficacy of i.c.v. delivery of rhPPT1 into the cerebrospinal fluid (CSF) in *Cln1^−/−^* mice. In addition to the well-characterized brain pathology in CLN1 disease, we recently highlighted the involvement of the spinal cord early in disease progression (15, 23). Therefore, we also compared the efficacy of i.t. delivery into the lumbosacral space and a dual delivery approach combining i.c.v. and i.t. infusions of the same total dose of enzyme to determine the best route of administration in *Cln1^−/−^* mice, before moving to test rhPPT1 in sheep.

Our data show that repeated dosing of rhPPT1 is an effective therapy in animal models of CLN1 disease across different species. These results represent a key step toward clinical testing of ERT in children with CLN1 disease.

## Results

### In vitro characterization and i.c.v. delivery of rhPPT1.

**Figure 1:**
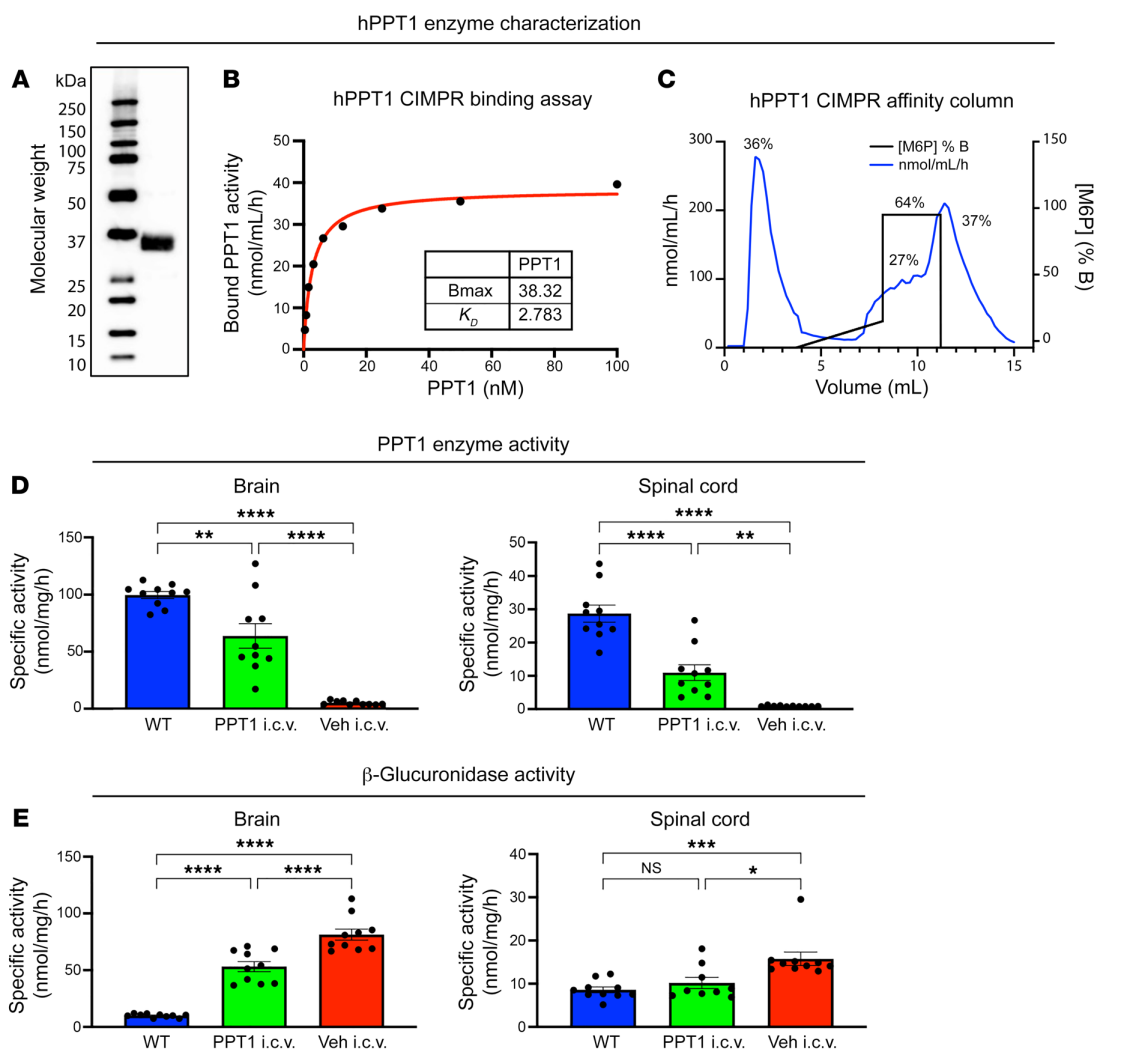
rhPPT1 enzyme characterization and in vivo efficacy in *Cln1^–/–^* mice. (**A**) Representative Western blot image of staining for PPT1 protein (~37 kDa) from CHO cell lysates (see full gel in Supplemental Figure 7). (**B** and **C**) rhPPT1 assay for binding to immobilized CI-MPR showing the (**B**) half-maximal binding (*K_D_*) of rhPPT1 at 2.78 nM and maximum binding (Bmax) at 38.32 nM and (**C**) that 64% of the loaded rhPPT1 remained bound to the affinity column. Specific activity in nmol/mg/h of (**D**) PPT1 and (**E**) β-glucuronidase enzymes from homogenates collected from mice 24 hours after their last i.c.v. infusion showing statistically significantly increased PPT1 activity and reduced β-glucuronidase activity in both the brains and spinal cords of treated mice (PPT1 i.c.v.) as compared with vehicle-treated controls (Veh i.c.v.). However, these enzyme values were not normalized to levels in WT control mice. Data represent the mean ± SEM; *n =* 10. *P < 0.05, **P < 0.01, ***P < 0.001, and ****P < 0.0001, by 1-way ANOVA with post hoc Bonferroni’s correction (see Supplemental Data File 2 for full *P* values).

The rhPPT1 used in this study was expressed and purified as previously described (17). Western blotting showed a molecular weight just below 37 kDa (Figure 1A), consistent with the previously reported migration of rhPPT1 as a 34 kDa band in an SDS-PAGE gel (17) (see full gel in Supplemental Figure 7). To ensure that this rhPPT1 preparation had properties appropriate for in vivo use, we determined additional biochemical characterizations of the enzyme. The half-maximal binding (*K_D_*) of rhPPT1 to the MPR was 2.8 nM (Figure 1B). Approximately 64% of the total rhPPT1 loaded onto a cation-independent mannose-6-phosphate receptor (CI-MPR) affinity column was retained on the column (Figure 1C). Analysis of site-specific glycosylation revealed that the asparagine residues Asn170, Asn185, and Asn205 were highly glycosylated. Asn170 contained 29% biphosphorylation and 15% monophosphorylation, Asn185 was 71% monophosphorylated, and Asn205 contained 23% biphosphorylation and 36% monophosphorylation. These biochemical properties were favorable for the binding and uptake of our rhPPT1 and its use in an ERT strategy.

We next tested the capability of monthly i.c.v. infusions of 20 μg rhPPT1 (5 μL of a 4 mg/mL solution) to increase the level of enzymatically active PPT1 in the CNS of *Cln1^−/−^* mice. This dose and infusion frequency in mice were chosen on the basis of our previous dose-response studies following a single i.t. infusion of rhPPT1 into *Cln1^−/−^* mice (16) and the relative persistence of PPT1 enzyme activity within the CNS (16).

In the current study, we collected brain and spinal cord tissue from mice 24 hours after the final infusion at 6 months. Delivery of rhPPT1 i.c.v. resulted in a statistically significant increase in PPT1 activity within the CNS of *Cln1^−/−^* mice when compared with vehicle-treated control mice, which had virtually undetectable levels of PPT1 activity (Figure 1D). This elevation in PPT1 activity was seen in both the brain (~64% of WT) and spinal cord (~38% of WT) of rhPPT1-treated mice. We previously showed that a reduction in the secondary elevation of other lysosomal enzymes that accompanies PPT1 deficiency can serve as a biochemical surrogate of therapeutic response in *Cln1^−/−^* mice (23, 24). Six-month-old vehicle-treated *Cln1^−/−^* mice had elevated levels of β-glucuronidase activity compared with WT controls. We found that β-glucuronidase activity was statistically significantly reduced in *Cln1^−/−^* mice receiving i.c.v. rhPPT1 infusions compared with their vehicle-treated counterparts (Figure 1E).

### Intracerebroventricular administration of rhPPT1 significantly improves motor performance in Cln1^−/−^ mice.

**Figure 2:**
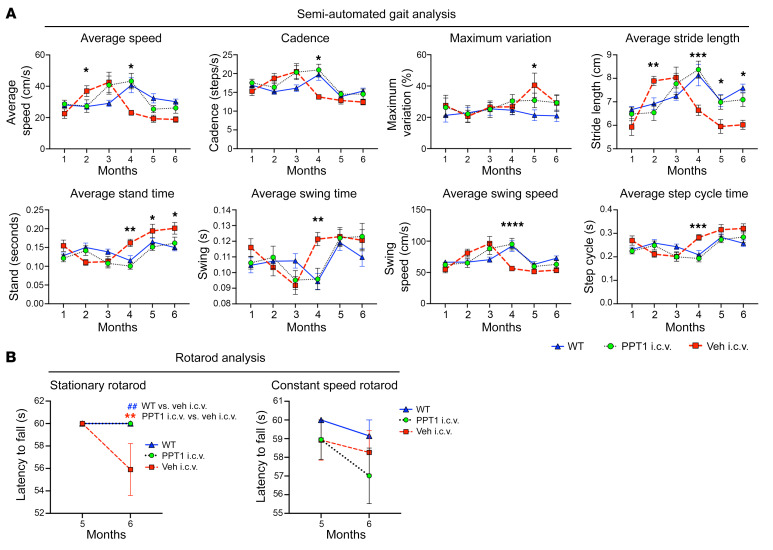
Improved motor performance in i.c.v. treated *Cln1^–/–^* mice. (**A**) Semiautomated gait analysis measures of average speed (cm/s), cadence (steps/second), maximum variation of speed (percentage), stride length (cm), standing time (s), swing time (s), swing speed (cm/s), and step cycle (s) from 1–6 months, showing an overall improved performance of mice treated i.c.v. with PPT1 (PPT1 i.c.v.) compared with mice treated i.c.v. with vehicle (Veh i.c.v.), and similar to WT values. (**B**) Stationary and constant speed rotarod tests in 5- and 6-month-old mice. The mice treated i.c.v. with PPT1 performed similarly to WT mice, whereas mice treated i.c.v. with vehicle had a statistically significant reduction in latency to fall (s) in the stationary rotarod test at 6 months. Both PPT1- and vehicle-treated mice showed a reduced latency to fall at 6 months in the constant speed rotarod test, but this did not reach statistical significance. Data represent the mean ± SEM; *n =* 10. *P < 0.05, **P < 0.01, ***P < 0.001, and ****P < 0.0001; ^##^*P* < 0.01 (WT vs. i.c.v vehicle-treated mice), by 2-way, mixed-effects ANOVA with post hoc Bonferroni’s correction (see Supplemental Data File 2 for full *P* values).

We first determined whether monthly i.c.v. infusions of the same 20 μg dose of rhPPT1 would ameliorate previously characterized behavioral phenotypes in *Cln1^−/−^* mice. Semiautomated gait analysis revealed that *Cln1^−/−^* mice treated i.c.v. with vehicle displayed an early period of hypermobility followed by a general decline in overall mobility starting at 4 months, with statistically significant effects on overall speed, cadence, and limb movement (Figure 2A). This performance was comparable to our previous gait analysis data from untreated *Cln1^−/−^* mice (15, 23). In contrast, the group treated i.c.v. with rhPPT1 had improved gait performance. We noted statistically significant differences between the i.c.v. rhPPT1–treated and i.c.v. vehicle–treated groups across most gait parameters, with a gait performance of i.c.v. rhPPT1–treated *Cln1^−/−^* mice that was more similar to that of WT controls (Figure 2A).

We previously demonstrated decreased rotarod performance of *Cln1^−/−^* mice as compared with WT controls beginning at 5 months of age (25, 26). Therefore, we also tested WT, i.c.v. rhPPT1–treated, and i.c.v. vehicle–treated *Cln1^−/−^* mice on both stationary and constant speed rotarod paradigms. In the constant speed rotarod test, both i.c.v. rhPPT1– and vehicle–treated groups showed impaired performance at 5 and 6 months compared with their WT counterparts (Figure 2B), but this was not statistically significant. In contrast, in the stationary paradigm, while i.c.v. vehicle–treated mice had a statistically significantly shorter latency to fall at 6 months (Figure 2B), i.c.v. rhPPT1–treated mice performed as well as their WT counterparts.

### Intracerebroventricular administration of rhPPT1 significantly attenuates Cln1^−/−^ neuropathology.

**Figure 3:**
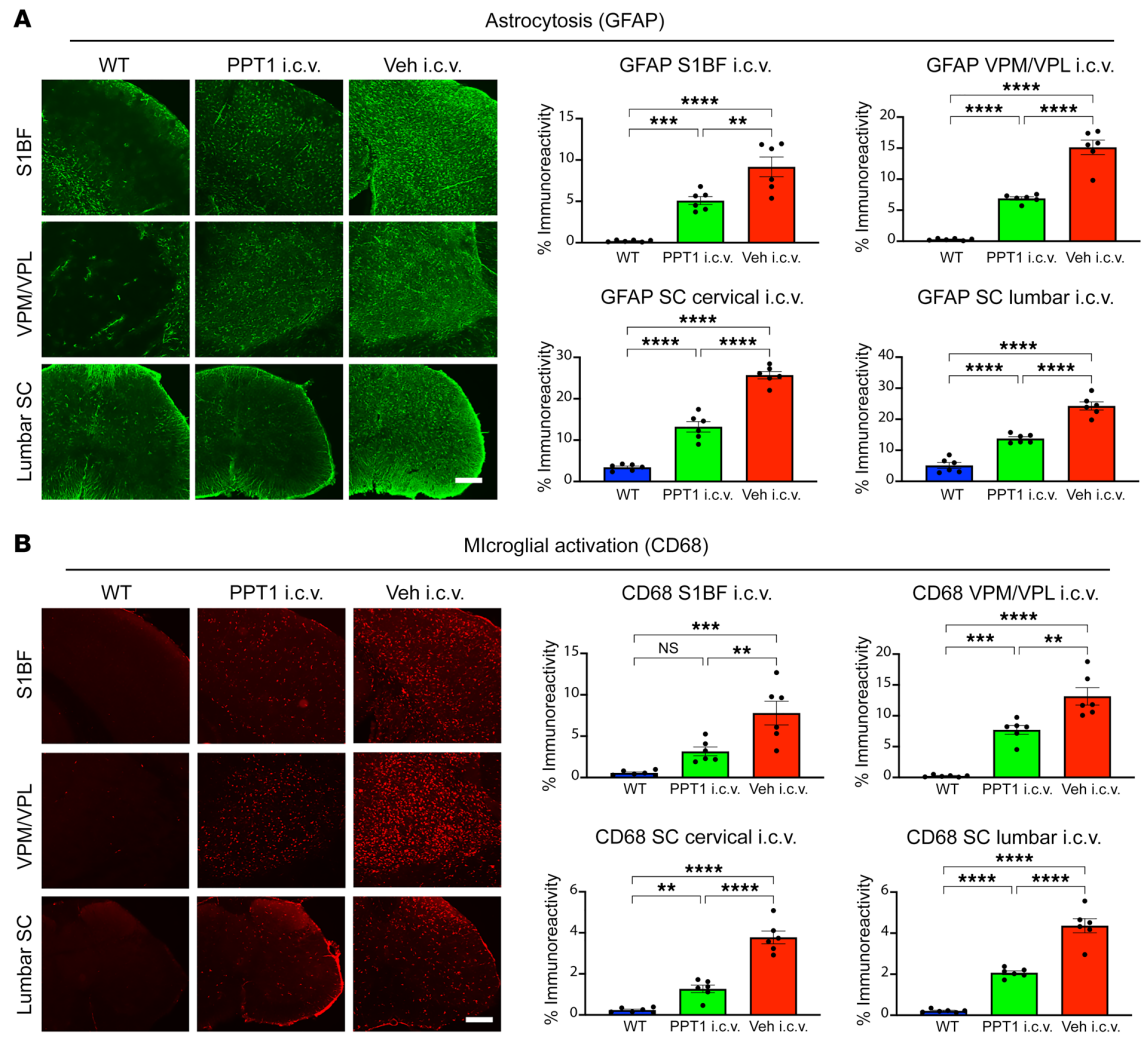
Decreased astrocytosis and microglial activation in the brains and spinal cords of i.c.v. treated *Cln1^–/–^* mice. Representative immunofluorescence images and thresholding image analysis showing an overall statistically significant reduction in (**A**) astrocytosis (GFAP) and (**B**) microglial activation (CD68) in i.c.v. treated (PPT1 i.c.v.) compared with vehicle-treated (Veh i.c.v.) mice across the S1BF, VPM/VPL, and cervical and lumbar spinal cord (SC). However, these did not reach WT levels across any of the regions except for CD68 in the S1BF. Scale bars: 100 μm. Data represent the mean ± SEM; *n =* 6. **P < 0.01, ***P < 0.001, and ****P < 0.0001, by 1-way ANOVA with post hoc Bonferroni’s correction (see Supplemental Data File 2 for full *P* values).

**Figure 4:**
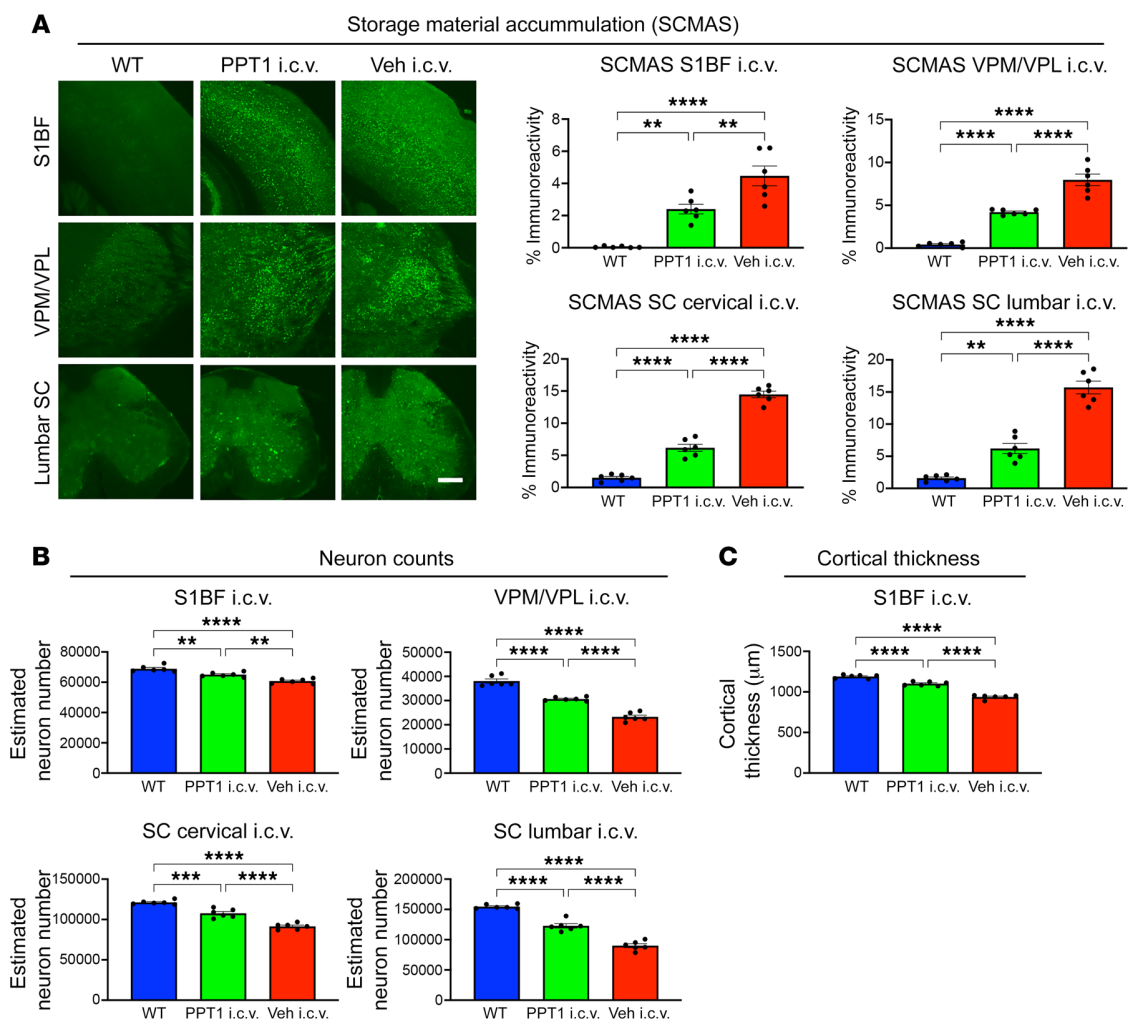
Decreased storage material accumulation, improved neuron survival, and cortical atrophy in i.c.v. treated *Cln1^–/–^* mice. (**A**) Representative immunofluorescence images and thresholding image analysis of SCMAS levels showing an overall statistically significant reduction in i.c.v. treated mouse brains and spinal cords compared with vehicle-treated mice across the S1BF, VPM/VPL, and SC. However, these did not reach WT levels across any of the regions. Scale bar: 100 μm. Statistically significant improvements in (**B**) neuron counts across all regions and (**C**) reduced cortical atrophy (S1BF) in PPT1-treated mice compared with vehicle-treated mice, but not completely normalized to WT values. Data represent the mean ± SEM; *n =* 6. **P < 0.01, ***P < 0.001, and ****P < 0.0001, by 1-Way ANOVA with post hoc Bonferroni’s correction (see Supplemental Data File 2 for full *P* values).

The brains and spinal cords of i.c.v. rhPPT1– and vehicle–treated mice were analyzed for neuropathological markers in well-established regions of known pathology. These included the primary somatosensory barrel field (S1BF), the ventral posterior nuclei of the thalamus (VPM/VPL), and the ventral horn of the cervical and lumbosacral spinal cord (14, 15). Vehicle-treated *Cln1^−/−^* mice had statistically significantly elevated levels of activated astrocytes (Figure 3A) and microglia (Figure 3B) across all CNS regions. These levels were statistically significantly reduced in i.c.v. rhPPT1–treated *Cln1^−/−^* mice (Figure 3, A and B). Similarly, the statistically significantly increased levels of intralysosomal subunit C of mitochondrial ATP synthase (SCMAS) present in all CNS regions in the vehicle-treated group were statistically significantly reduced in the i.c.v. rhPPT1–treated group (Figure 4A). Statistically significant neuron loss and cortical atrophy are observed in the brains and spinal cords of *Cln1^−/−^* mice at end stage (14, 15, 23, 27) and were also seen in i.c.v. vehicle–treated *Cln1^−/−^* mice, with statistically significantly fewer neurons in the brain and spinal cord. In contrast, i.c.v. rhPPT1–treated mice showed statistically significantly reduced neuron loss across all regions (Figure 4B). These i.c.v. rhPPT1–treated mice also showed statistically significantly less cortical atrophy (Figure 4C) compared with the i.c.v. vehicle–treated controls.

### Alternate delivery routes are not as effective as i.c.v. delivery alone.

The spinal cord is severely affected in *Cln1^−/−^* mice, starting in the early stages of disease progression (15, 16, 23). Therefore, we also tested whether delivering monthly i.t. injections of the same 20 μg dose of rhPPT1 to *Cln1^−/−^* mice would improve the treatment outcomes compared with i.c.v. infusions alone. We also tested a dual delivery strategy by infusing the same total 20 μg dose of rhPPT1 via both the i.c.v. and i.t. routes, with half the dose (10 μg or 2.5 μL 4 mg/mL rhPPT1) delivered via each route.

For both i.t. rhPPT1–treated mice as well a combination of i.c.v. and i.t. rhPPT1 deliveries, we observed a statistically significant increase in PPT1 enzyme activity in the brains and spinal cords of *Cln1^−/−^* mice (Supplemental Figure 1A; supplemental material available online with this article; https://doi.org/10.1172/JCI163107DS1) and a decrease in β-glucuronidase activity (Supplemental Figure 1B) as compared with vehicle-treated controls. Mice treated i.t. with rhPPT1 outperformed i.t. vehicle–treated *Cln1^−/−^* mice at 6 months on the stationary rotarod and at 5 and 6 months on the constant-speed rotarod (Supplemental Figure 2B). However, dual delivery of rhPPT1 via both i.c.v. and i.t. routes did not show statistically significant treatment effects in either rotarod test (Supplemental Figure 2B and Supplemental Figure 3B). Furthermore, compared with i.c.v. rhPPT1–treated mice, both i.t. delivery alone and dual delivery of rhPPT1 had less effect on the gait performance of *Cln1^−/−^* mice (Supplemental Figure 2A and Supplemental Figure 3A).

Using neuropathological outcome measures, i.t. administration of rhPPT1 and dual delivery rhPPT1-treated *Cln1^−/−^* mice displayed a variety of treatment effects, some of which were statistically significantly different from those seen in the vehicle-treated control mice. These effects varied across CNS regions and varied for astrocytosis (Supplemental Figure 4A), microglial activation (Supplemental Figure 4B), storage material accumulation (Supplemental Figure 5A), neuron survival, and cortical thickness (Supplemental Figure 5B). However, neither of these alternative delivery routes had a level of rescue across all pathological phenotypes comparable to that provided by i.c.v. administration of rhPPT1 alone.

### Intracerebroventricular administration of rhPPT1 in CLN1^R151X^ sheep.

We next sought to scale up the repeated i.c.v. rhPPT1 dosing strategy in a larger and more complex CNS. We first confirmed that a single dose of rhPPT1 would elevate PPT1 activity in the CNS of the recently generated *CLN1^R151X^* sheep model (20). Four 6-month-old homozygous *CLN1^R151X^* sheep were infused with a single dose of rhPPT1. The scaled up dose of 4 mg rhPPT1 (1 mL total volume of the same 4 mg/mL enzyme preparation used in the *Cln1^−/−^* mice) was based on practical considerations of the volume and rate of infusion, while minimizing the anesthetic risk, and was delivered using infusion parameters similar to those used when delivering ERT to tripeptidyl peptidase 1–deficient (TPP1-deficient) dogs (21, 22).

Sheep that received a single 4 mg dose of i.c.v. rhPPT1 were sacrificed 24 hours, 1 week, 2 weeks, or 1 month after the infusion. PPT1 activity in the CSF was found to be increased to 92% of WT activity 24 hours after administration. Thereafter, it dropped to 7%, 3%, and 3% at 1 week, 2 weeks, and 1 month after dosing, respectively. At 24 hours, PPT1 activity was elevated across multiple brain regions including the subventricular zone (12.6% of WT), cortex (1.1% of WT), midbrain (0.9% of WT), and brainstem (2.9% of WT). This level of PPT1 activity progressively decreased in animals sacrificed 1 week, 2 weeks, or 1 month after infusion (Supplemental Figure 6).

To test the therapeutic efficacy of i.c.v. rhPPT1, 6-month-old (early symptomatic) homozygous *CLN1^R151X^* sheep (20) (*n =* 2) received over 7 months a monthly i.c.v. infusion of 4 mg rhPPT1 from the same batch of enzyme as that used in *Cln1^−/−^* mice (1 mL of 4 mg/mL for each infusion), with the same infusion ports and parameters previously used when delivering rhTPP1 to CLN2-deficient dogs (21, 22). The rhPPT1-treated *CLN1^R151X^* sheep were sacrificed at 13 months of age, along with 2 age-matched WT and 2 untreated *CLN1^R151X^* sheep. The CSF levels in the rhPPT1-treated sheep showed increases of up to 150% of WT PPT1 enzyme activity levels 1 week after the final administration of rhPPT1 (Supplemental Figure 6).

**Figure 5:**
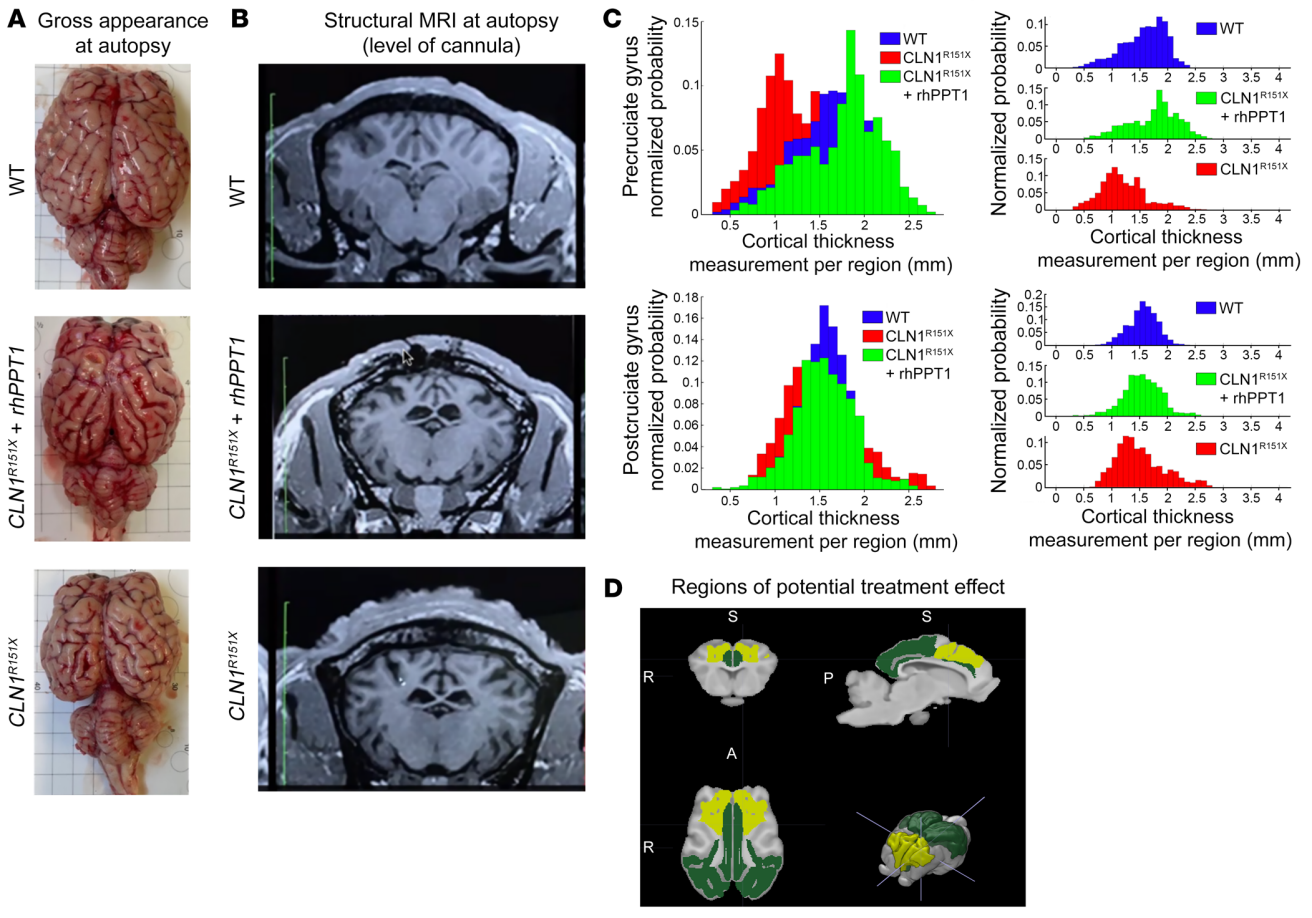
Therapeutic effect of i.c.v. administration of rhPPT1 in *CLN1^R151X^* sheep. (**A**) Gross anatomical examination and (**B**) structural MRI analysis showed a positive treatment effect of i.c.v. administration of rhPPT1 (*CLN1^R151X^* + rhPPT1) compared with untreated *CLN1^R151X^* sheep and WT controls showing an overall reduction in cerebral and cerebellar atrophy in rhPPT1-treated *CLN1^R151X^* sheep. (**C**) Histograms of individual measures of cortical thickness in the pre- and post-cruciate gyri showing the extent of treatment effect in these regions, with the movement of values in rhPPT1-treated *CLN1^R151X^* sheep (green) moving closer to those for WT sheep (blue) than for untreated *CLN1^R151X^* controls (see Supplemental Table 1 and Supplemental Data File 1 for all cortical thicknesses). (**D**) Solid 3D representation showing colored INRA ovine atlas (24) cortical regions in which a significant treatment effect upon individual thickness measurements was detected. ANOVA (ERT > untreated, *P <* 0.0001). Yellow and green colors indicate the magnitude of this effect, with no significant treatment effect detected in the gray cortical regions (subcortical structures not analyzed). Yellow indicates regions where the mean cortical thickness values for rhPPT1-treated *CLN1^R151X^* sheep were closer to WT values (greater treatment effect), and green represents regions in which these values were closer to those of untreated *CLN1^R151X^* sheep (positive treatment effect).

All sheep underwent structural MRI of the brain within 1 hour of sacrifice, following removal of the metal ports used for transfusions. Cortical gray matter (GM) regions undergo differential degrees of atrophy in *CLN1^R151X^* homozygous animals (20). Gross anatomical examination of brains collected at autopsy revealed pronounced cerebral and cerebellar atrophy in the untreated *CLN1^R151X^* sheep compared with WT sheep brains. In contrast, rhPPT1-treated *CLN1^R151X^* sheep brains showed less atrophy of both the forebrain and cerebellum (Figure 5A). MRI analysis showed a similar protective effect of rhPPT1 administration in rhPPT1-treated *CLN1^R151X^* sheep (Figure 5B). To assess the relative preservation of cortical regions, even in the presence of underlying GM atrophy, we normalized regional GM volumes to total GM volume (summarized in Supplemental Data File 1 and Supplemental Table 1). After atlas-based segmentation, histograms of the thickness of individual cortical regions were compared across experimental groups in each of 18 neocortical structures from the Institut National de la Recherche Agronomique (INRA) atlas (28) (Supplemental Data File 3). This revealed a range of positive treatment effects on cortical thickness in most cortical regions, with a shift in thickness histograms closer to WT values in the rhPPT1-treated *CLN1^R151X^* sheep (Figure 5C, with data from all regions in Supplemental Data File 3). These effects were not uniform across all cortical regions (Figure 5D), being more pronounced rostrally than in caudal cortical areas, with a few regions not benefitting from rhPPT1 treatment (data from all regions summarized in Supplemental Data File 1 and Supplemental Table 1).

**Figure 6:**
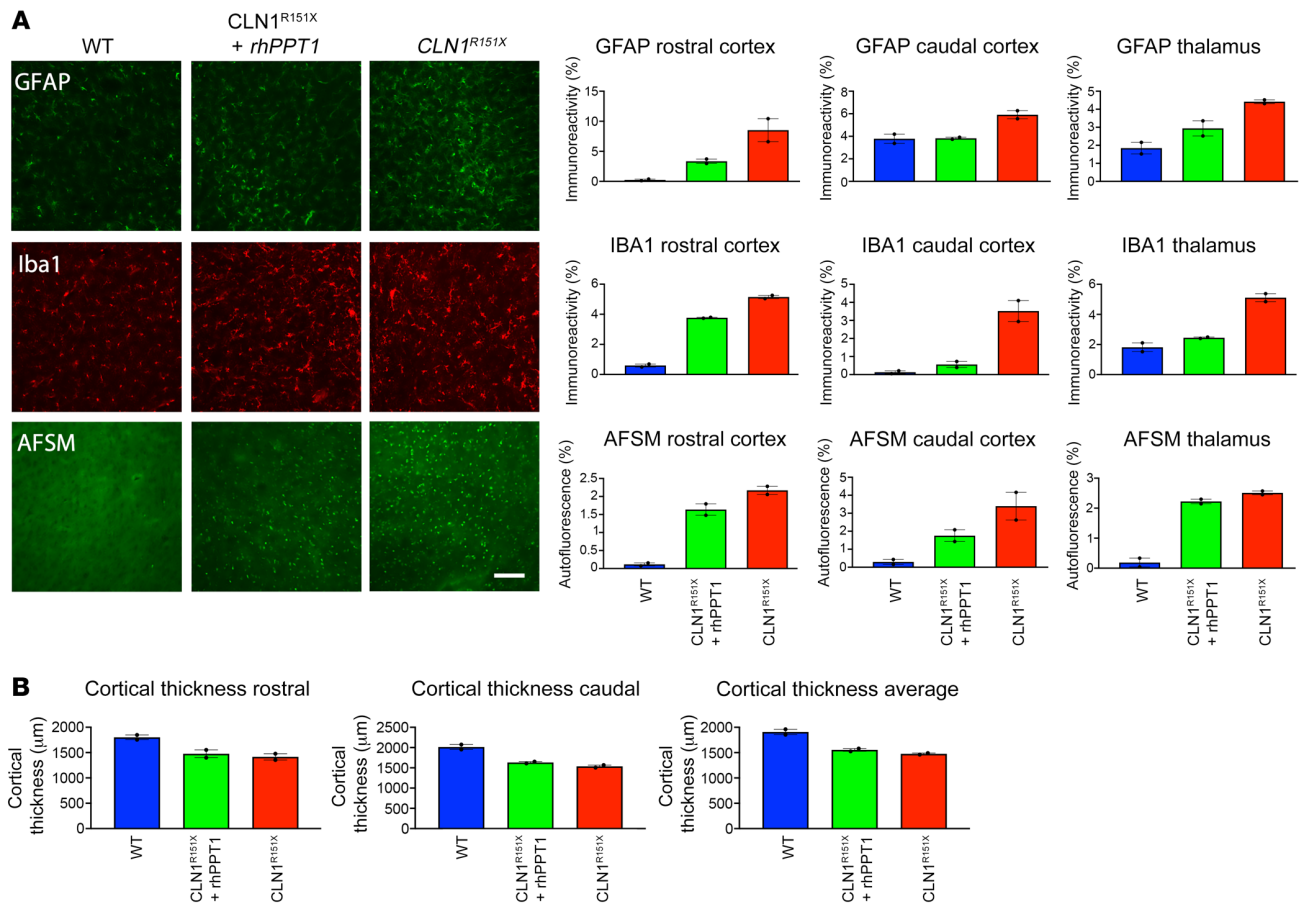
Therapeutic effect on neuropathology of i.c.v. administration of rhPPT1 to *CLN1^R151X^* sheep. (**A**) Representative immunofluorescence images of the cortex and thresholding imaging analysis (*n =* 2) of WT, i.c.v. rhPPT1–treated (*CLN1^R151X^* + rhPPT1), and untreated *CLN1^R151X^* sheep showing a reduction in markers for astrocytosis (GFAP), microglial activation (Iba1), and AFSM accumulation across the rostral and caudal regions of the somatosensory cortex as well as the thalamus. A reduction was seen across all the markers in rhPPT1-treated *CLN1^R151X^* sheep compared with untreated *CLN1^R151X^* sheep, although these did not reach WT levels. Scale bar: 50 μm. (**B**) Measurements of cortical thickness in the rostral and caudal somatosensory cortices (and averaged values) showing slightly increased values in rhPPT1-treated *CLN1^R151X^* sheep compared with untreated *CLN1^R151X^* sheep. Data represent the mean ± SEM.

We also performed a neuropathological analysis of WT sheep, untreated *CLN1^R151X^* sheep, and rhPPT1-treated *CLN1^R151X^* sheep brains. We analyzed previously identified affected brain regions (20) including the primary somatosensory cortex, at both the level of the i.c.v. catheter (located in the rostral somatosensory cortex) and of the thalamus (the caudal somatosensory cortex), in addition to the thalamus itself. We observed an overall reduction in astrocytosis, microglial activation, and autofluorescent storage material (AFSM) accumulation (Figure 6A) in the rhPPT1-treated *CLN1^R151X^* sheep compared with the untreated *CLN1^R151X^* sheep. The thickness of the primary somatosensory cortex was moderately increased in the rhPPT1-treated *CLN1^R151X^* sheep as compared with the untreated *CLN1^R151X^* sheep (Figure 6B). This finding correlated with our gross anatomical and imaging observations (Figure 5).

## Discussion

Devising an effective treatment strategy for CLN1 disease is a particularly difficult challenge. In addition to its early onset and rapid progression, this disorder affects widespread and anatomically distant regions of the CNS, all of which need treatment. CLN1 disease is theoretically amenable to gene therapy via CNS delivery of the *CLN1* gene or, in ERT, via CNS delivery of recombinant PPT1 enzyme (3, 7). Although gene therapy for CLN1 disease has shown promise in preclinical studies, its effects are relatively localized, and, compared with its use in CLN2 disease, it is less effective (23). At present, there remain limitations for the immediate clinical translation of gene therapy including transduction efficiency, immune response to viral vectors, and appropriate routes of administration to maximize biodistribution (29–31), and work is ongoing to overcome these obstacles. We therefore reasoned that ERT, with its potential to treat the PPT1-deficient CNS, could represent a means to rapid and potentially more effective translation, despite potential challenges such as the need for repeated enzyme delivery, the attendant risk of port-associated infections, and potential immune responses to the exogenous protein (3, 32, 33). Our data show that repeated dosing of rhPPT1 can serve as an effective enzyme replacement strategy for CLN1 disease, with demonstrable efficacy across both small and large animal models of this fatal disorder.

Having prepared rhPPT1 with favorable uptake properties, the key to the success of our ERT strategy for CLN1 disease was defining the method for delivering this enzyme. The enzyme needs to be administered in sufficient amounts to improve disease outcomes in both the brain and spinal cord (15, 23). Such enzyme administration to the CSF could plausibly be achieved by either i.c.v. or i.t. delivery. We reasoned that dosing rhPPT1 i.t. would be a suitable means of treating the significant spinal pathology that occurs in *Cln1^−/−^* mice (15, 16, 23). While i.c.v. infusions would treat affected brain regions, they potentially may not effectively reach the spinal cord, although our data suggest that i.c.v. delivery did in fact reach the lumbar spinal cord of the sheep relatively quickly (Supplemental Figure 6B). Nevertheless, on the basis of our previous gene therapy study in mice (23), we opted to test the potential of either route of administration, comparing i.c.v. and i.t. rhPPT1 delivery in mice, in addition to combined administration via both routes. Our data show that, regardless of the route of delivery, repeated administration of rhPPT1 to the CSF was well tolerated in *Cln1^−/−^* mice. However, compared with i.c.v. delivery, there was less overall therapeutic benefit in the brain after i.t. rhPPT1 delivery. Splitting the same total dose of rhPPT1 across both delivery routes was also less effective by all outcome measures, suggesting that a certain threshold of rhPPT1 activity must be reached for ERT to be effective. Taken together, our *Cln1^−/−^* mouse data revealed that i.c.v. administration alone provided rhPPT1 biodistribution that was sufficient to reach both the brain and spinal cord. This is consistent with the greater efficacy of i.c.v. rhPPT1 infusions in *Cln1^−/−^* mice according to both motor performance and pathological outcome measures.

Although mouse models provide a valuable testing ground for preclinical strategies, many promising approaches fail to be translated into successful clinical treatments. This is not surprising, given the considerable differences in the relative complexity and physical size of the CNS between mice and humans, in addition to considerable species differences in drug metabolism (6). Large animal models represent a crucial intermediate test system that is well suited to determine how to adapt drug delivery and dosing to successfully treat a larger and more complex CNS. This approach was successful in the preclinical testing of i.c.v. enzyme replacement therapy for CLN2 disease, the late-infantile form of NCL (22, 34, 35). This led to the first FDA-approved disease-limiting therapy for any neuronopathic LSD (6). Such preclinical studies in either dogs or sheep have mostly relied on naturally occurring mutants. However, the advent of modern gene editing methods such as CRISPR/Cas9 allowed us to generate a *CLN1^R151X^* sheep model with a common human disease–causing mutation (20). This model was specifically created for the purpose of testing how to scale up preclinical strategies that show efficacy in *Cln1^−/−^* mice for human translation. This sheep model also allowed us to test outcome measures that might feasibly be used in a subsequent human clinical trial. We are still developing suitable neurological outcome assessment measurements for use in *CLN1^R151X^* sheep (20), including maze testing of cognitive function and other neurological tests similar to those performed in naturally occurring NCL sheep models (36, 37). However, our data revealed that quantitative MRI could detect treatment effects in sheep using a widely available human clinical MRI system with a standard clinical sequence and a widely used and validated human post-processing approach. This is encouraging for the subsequent use of such an imaging methodology in children with CLN1 disease (38).

The demonstrable efficacy of rhPPT1 across both mouse and sheep models of CLN1 disease indicates the value of such a cross-species approach for developing a successful treatment strategy. While this study was focused on a single rare disorder, this approach has broader applicability for developing therapies for a range of similar conditions. ERT with rhPPT1 successfully attenuated disease progression in *Cln1^−/−^* mice and *CLN1^R151X^* sheep. As early diagnosis of CLN1 disease becomes more feasible, having an effective means to intervene therapeutically would completely alter clinical practice. In order to arrive at complete “normalization” of disease, it may be necessary to achieve a sustained and higher concentration of PPT1 in the CNS. Increasing the frequency of enzyme infusions or administering a higher dose of enzymes could accomplish this, at least acutely, in *Cln1^−/−^* mice (Supplemental Figure 1C), and it will be important to test such strategies in chronic treatment studies. Alternatively, the cellular uptake of PPT1 could be further improved by engineering a highly phosphorylated version, as has been shown in vitro for other lysosomal enzymes (39). It is also possible that initiating treatment earlier, using recombinant sheep PPT1 or an alternate cell line to produce enzymes may further improve efficacy in *CLN1^R151X^* sheep. In addition, it will also be important to treat other affected organs such as the eye and other sites outside the CNS, which are unlikely to be treated effectively by i.c.v. delivery of rhPPT1.

Overall, we have shown that repeated dosing of ERT to treat CLN1 disease can be a viable therapeutic strategy and that the cross-species pipeline with defined outcome measures will facilitate dosing and biodistribution studies. Together, these represent what we believe to be a significant step forward in the development of a clinical therapy for this fatal disease that currently lacks an FDA-approved treatment.

## Methods

### Study design.

The goal of this study was to test the efficacy of repeated delivery of 20 μg rhPPT1 enzyme to the CNS of PPT1-deficient mice (*Cln1^−/−^*) and sheep (*CLN1^R151X^*). First, *Cln1^−/−^* mice were given monthly infusions of rhPPT1 enzyme starting at P1 via the i.c.v., i.t., or combined i.c.v. plus i.t. (split dose) routes for 6 months, with appropriate controls — vehicle-treated *Cln1^−/−^* mice and naive WT mice. Intracerebrovascular infusions from P30 were done via a chronic cannula placed in the left ventricle. Mice were assessed at monthly intervals for changes in motor performance using semiautomated gait analysis and rotarod paradigms. At 6 months (end-stage disease), the mice were sacrificed and the collected tissue analyzed for enzymatic activity and neuropathology. All analyses were performed in a blinded manner with regard to genotype and treatment. Having established the most effective route of rhPPT1 administration in mice, *CLN1^R151X^* sheep, starting at 6 months of age, were given monthly infusions of rhPPT1 or vehicle for 7 months via a surgically implanted i.c.v. cannula. At 13 months, the sheep were sacrificed and subjected to MRI within 1 hour of sacrifice, and tissue was subsequently collected for biochemical and histopathological analysis. An acute dose-response study was also performed in 2-month-old *Cln1^−/−^* mice to determine whether delivering a higher, 40 μg, dose of rhPPT1 would further increase CNS levels of PPT1 activity above those seen with a 20 μg dose.

### Animals.

Congenic *Cln1^−/−^* and WT mice were previously engineered at the University of Texas Southwestern Medical Center in Dallas, Texas (19), and were maintained on a C57Bl/6J background. *CLN1^R151X^* sheep were previously engineered at the Roslin Institute, University of Edinburgh, Easter Bush, Scotland, United Kingdom (20), and were now generated by breeding heterozygous sheep. Homozygous *CLN1^R151X^* sheep were confirmed by appropriate genotyping (20). See Supplemental Methods for further details.

### rhPPT1 enzyme formulation and administration.

The rhPPT1 used in this study is identical to that previously produced and purified using Chinese hamster ovary (CHO) cells as described previously (17) and characterized by Western blotting. Purified PPT1 was tested by a CI-MPR assay for binding affinity to the immobilized CI-MPR, and the half-maximal binding (*K_D_*) was determined as described before (17). Glycopeptide composition (glycosylation and phosphorylation) was determined by liquid chromatography high-resolution UV mass spectrometry (LC-UV-HRMS), as described previously (17). All mice, regardless of route of administration (i.c.v., i.t., or i.c.v. + i.t.), received the same total dose of enzyme — 5 μL rhPPT1 at 4 mg/mL — as monthly infusions for 6 months. Six-month-old *CLN1^R151X^* sheep received 1 mL of the same 4 mg/mL rhPPT1 preparation, infused at a rate of 0.6 mL/h. These infusions were given monthly starting at 6 months and ending at 13 months of age. See Supplemental Methods for further details.

### Murine behavioral analysis.

Rotarod testing using stationary (60 s) and constant speed (2.5 rpm) paradigms (23) and gait analysis using the CatWalk XT system (Noldus Information Technology) were performed as previously described (15) using 10 mice per group at monthly intervals, beginning at 1 month until 6 months. All behavioral tests were performed after appropriate habituation and training. See Supplemental Methods for further details.

### Ovine MRI analysis.

Sheep were imaged within 1 hour of being sacrificed. Structural sequences included T1- and T2-weighted 3D imaging and fluid-attenuated inversion recovery (FLAIR) imaging with whole brain coverage. Apparent diffusion coefficients were calculated using readout segmentation of long variable echo-trains (RESOLVE) to minimize echoplanar distortions. The extracted brains were classified using the advanced normalization tools (ANTs) for 3 tissue classes (CSF, GM, and white matter [WM]). Extracted brains were then coregistered with the INRA brain-only template (28). Total brain volume was calculated from a combination of the GM and WM tissue class probability maps. All metrics were adjusted for total intracranial volume (GM + WM + CSF), total brain volume (GM + WM), and total GM volume. We have previously described that areas of cortical GM undergo differential degrees of atrophy in homozygous *CLN1^R151X^* sheep (20), and therefore regional GM volumes were normalized to total GM volume to assess for the relative preservation of cortical regions. See Supplemental Methods for further details.

### Tissue collection and histopathological analysis.

Mouse brain and spinal cord tissues were collected for biochemical and histological processing as previously described (23). Sheep tissues were collected for biochemical analyses and histological processing as previously described (20). Mouse brains and spinal cords were stained for cresyl fast violet as well as immunostained for glial markers and storage material as previously described (15). Sheep brain sections were stained using a free-floating immunofluorescence protocol for astrocytes (glial fibrillary acidic protein [GFAP]) and microglia (ionized calcium-binding adapter molecule 1 [Iba1])], or left unstained to directly visualize AFSM (see Supplemental Methods for further details). Cresyl fast violet–stained tissue was analyzed for cortical thickness and neuron counts as described before (14, 15), and immunostained tissue and tissue for AFSM were analyzed using thresholding image analysis as described previously (15) (see Supplemental Methods for further details).

### Analysis of enzyme activity.

Mouse and sheep tissues were homogenized and analyzed for PPT1 activity using the 4-MU-palmitate fluorometric assay and normalized to total protein. Secondary elevations of another lysosomal enzyme, β-glucuronidase, were determined using the 4-MU-β-d-glucuronide fluorometric assay and normalized to total protein as previously described (23, 40).

### Statistics.

All measurements for histological processing were performed with the researcher blinded to genotype. All statistical analyses were performed using GraphPad Prism (GraphPad Software). Gait and rotarod data were analyzed using a 2-way ANOVA with post hoc Bonferroni’s correction, and all biochemical and histological analyses were done using a 1-way ANOVA with post hoc Bonferroni’s correction. In all instances, a *P* value of 0.05 or less was considered significant.

### Study approval.

All procedures were performed in accordance with NIH guidelines under a protocol approved by the IACUC of Washington University School of Medicine (protocols 2018-0215 and 21-0292). Sheep studies were reviewed and approved by the Animal Welfare and Ethical Review Board (AWERB) of the Roslin Institute and conducted under the authority of the UK Home Office (equivalent of an IACUC). The work detailed here was carried out under license number PEFC7DB6A.

## Author contributions

JDC, TMW, SE, MSS, and HRN designed the study. JYL and SLH prepared the enzyme. ACP and SLE performed enzyme Western blotting. NS was responsible for biochemical characterization of the enzyme. HRN, SHW, EME, KT, SQL, RN, and JTD performed in vivo mouse studies, behavior tests, biochemistry, and histology. SLE, GT, FM, PMB, MG, SG, PT, RG, EC, JN, CP, SG, SGL, CBAW, and MSS performed in vivo sheep studies and biochemistry. HRN, SHW, and KT performed sheep histology. JDC, SE, ACP, TMW, and MSS acquired funding. JDC, SE, TMW, and MSS were responsible for project administration. JDC, MSS, PID, TMW, and SE supervised the study. HRN, JDC, SE, and ACP wrote the original draft of the manuscript. TMW, MSS, PID, and SE, reviewed and edited the manuscript with input from all authors. HRN and SLE are co–first authors. The order of first authorship was assigned on the basis of overall contributions and writing of the manuscript.

## Supplementary Material

Supplemental data

Supplemental data set 1

Supplemental data set 2

Supplemental data set 3

## Version 1 08/30/2022

In-Press Preview

## Version 2 10/17/2022

Electronic publication
