# Supplementary material for: Cross-species efficacy of enzyme replacement therapy for CLN1 disease in mice and sheep
Source: J Clin Invest. 2022 Oct 17;132(20):e163107. doi: 10.1172/JCI163107 (PMC9566914; doi:10.1172/JCI163107)
Supplement: Supplemental data [file jci-132-163107-s160.pdf]

Supplementary Materials and Methods

Animals

Cln1$^{-/-}$ mice were generated by Gupta et al. at the University of Texas Southwestern Medical Center, Dallas, TX (1). Congenic *Cln1*$^{-/-}$ and wild-type (WT) mice were maintained on a C57Bl/6J background through homozygous mating of each colony. Animals were housed in an animal facility at Washington University School of Medicine in St. Louis under a 12-hour light/dark cycle. Food and water were provided *ad libitum*. All procedures were performed in accordance with NIH guidelines under a protocol approved by the Institutional Animal Care and Use Committee (IACUC) at Washington University School of Medicine. Unless otherwise stated, n=10 mice of each genotype and treatment group were used for gait analysis and rotarod testing and n=6 (3 male, 3 female) were used for biochemical and histopathological analyses. *CLN1*^{R151X} sheep were previously generated at the Roslin Institute, University of Edinburgh, Easter Bush, Scotland, UK and were generated by breeding heterozygous sheep. Homozygous *CLN1*^{R151X} sheep were confirmed by appropriate genotyping (2).

Four male homozygote *CLN1*^{R151X} sheep received a single ICV dose of 1 ml rhPPT1. Animals were sacrificed at 24 hours, 1 week, 2 weeks and 1 month post injection to determine the distribution and persistence of PPT1 activity. Three age-matched wild-type sheep from the same flock and birth cohort were used as controls. Two female *CLN1*^{R151X} sheep received repeated rhPPT1 ICV infusions from 6 months to 13 months of age – a total of 7 infusions for one sheep and 6 for the other. Three age-matched homozygote male and two wild-type females were used as positive and negative controls respectively.

rhPPT1 Enzyme characterization and administration

Formulation

rhPPT1 was produced and purified using Chinese Hamster Ovary (CHO) cells as previously described (3).

CI-MPR column chromatography

rhPPT1 sub-population separation and relative mannose-6-phosphate quantification was conducted through the loading of purified protein onto a soluble cation independent M6P receptor (sCI-MPR) affinity column, followed by an enzymatic activity assay. Roughly 1 µg of rhPPT1 was passed over 1 mL pre-conjugated resin, allowing the enzyme to bind. A 0-1 mM M6P gradient, over 4.5 column volumes (CVs), followed by a 3 CV step-elution (5 mM M6P) was used to elute any bound rhPPT1. Resultant fractions were tested for PPT1 activity and three rhPPT1 subpopulations were found; unbound rhPPT1 (no M6P), early elution (mono-phosphorylated), and late elution (bis-phosphorylated).

Glycopeptide analysis by mass spectrometry

Roughly 20 µg of purified PPT1 sample was denatured with 1.5M urea and 50mM ammonium carbonate, reduced with 25mM dithiothreitol, alkylated with 50mM iodacetamide, and finally digested with a 1:10 ratio of PPT1 to Lys-C and Trypsin (2 µg of each). Following the addition of up to 1% final concentration of formic acid, each sample was dried in a SpeedVac and reconstituted in 0.1% formic acid in water. Samples were then subject to either PNGase or EndoH glycan release and subsequent sample cleanup through the use of a HiPPR detergent removal column. Glycopeptides were characterized by liquid

chromatography high resolution mass spectrometry (LC-UV-HRMS) by loading processed PPT1 samples onto a Halo Peptide ES-C18 analytical column. A Thermo Scientific Dionex Ultimate 3000 UHPLC focused liquid chromatography system was operated using a flow rate of 25 µl/min and the eluent was composed of mobile phase A (0.1% formic acid in LC-MS grade water) and mobile phase B (0.1% formic acid in LC-MS grade acetonitrile). Glycopeptides were separated using a mobile phase B gradient of 1 to 50% over the course of 100 minutes. This was extended to 95% mobile phase B for 10 minutes in order to elute other highly retentive peptides from the column. The mass spectrometry analysis was carried out in tandem on a Orbitrap Fusion Tribrid system using the positive electrospray ionization mode, operated at a resolution of 120,000 at m/z = 200. Mass isolation was performed in the quadrupole in the scanning range of 775-1975 m/z. The MS raw data were analyzed by the peak list-generating software Genedata Expressionist. For analysis the PPT1 sequence was queried based on a tryptic digest, including a personalized N-Glycan library. This library includes the individual glycopeptide massed and therefore can be used to find matches in the raw data.

CI-MPR binding assay

Purified PPT1 was tested by CIMPR binding assay for affinity to the immobilized cation independent mannose-6-phosphate receptor (CI-MPR). 96-well high-binding polystyrene EIA plates were coated with 300ng/well of purified soluble IGF2/CI-MPR in 50mM sodium phosphate (pH 7.5), 150mM NaCl buffer for 1 hour. The coated plate was then blocked with 2% BSA in PBS for 1 hour and then incubated with 8.5 ng/well purified PPT1, 85 ng/well of purified PPT1, or binding buffer at 37°C for 30 minutes. After washing, a 4-MU standard curve and PPT1 activity reaction mix (20mM sodium citrate (pH 4.0), 50 mM NaCl, 0.02% Triton X-100, 5mM DTT, 2U/mL B-Glucosidase, 50 µM 4-methylumbelligeryl α-D-

glucopyranoside substrate) are applied to the plate and the plate is incubated at 37°C for 1 hour. The reaction is stopped with 125 µL 1M glycine stop buffer and fluorescence read using an excitation wavelength of 370 nm and an emission wavelength of 460 nm. Activity assay was used to determine Bmax and Kd of purified PPT1 samples to immobilized CI-MPR.

Western Blotting

Purified PPT1 (100 ng) was loaded and separated on a Bolt 4-12% Bis-Tris SDS-PAGE gel along with Bio-Rad Precision Plus WesternC prestained molecular weight markers (Bio-Rad #1610376; 2 µl per lane) at 150V for 45 minutes. Separated proteins were then transferred to a PVDF membrane using an iBlot Gel Transfer system (Thermo Scientific) per manufacturer instructions. The membrane was then blocked in 5% milk in PBS + 0.1% Tween-20 (PBST), then probed overnight with anti-PPT1 antibody (Atlas Antibodies #HPA021546; 1:1,250 in 5% milk in PBST). The next day, the blot was washed three times in PBST, then probed with anti-rabbit-IgG-HRP (Cytiva #NA934V; 1:5,000 in 5% milk in PBST) along with StrepTactin-HRP (Bio-Rad # 1610381; 1:10,000) to develop the molecular weight markers. After washing an additional three times in PBST, and once in PBS, the membrane was developed in ECL reagent (Cytiva RPN2232) for 5 minutes, then scanned with an ImageQuant LAS 4000 system (Cytiva).

rhPPT1 administration in Mice

All treatment groups received a total dose of 20µg of rhPPT1 (5 µl at a concentration of 4mg/ml), with control groups receiving the same volume of artificial Ringers vehicle (Thermo Fisher Scientific, MA). Wild-type mice remained uninjected. At postnatal day 1 (P1), *Cln1*$^{-/-}$ pups were anesthetized by hypothermia on an ice-pack at 4°C and received an

ICV infusion of 5μl rhPPT1 or vehicle into the left ventricle using a 0.5 ml syringe with 30-gauge needle. After weaning (P21), animals receiving ICV infusions had a sterile guide cannula (Plastics One, 2.4-mm cut depth, 26 gauge) stereotactically implanted into the left lateral ventricle, as before (4). Briefly, this was done using the following coordinates: 0.3 mm caudal to the bregma, 1 mm lateral to the midline, and 2.4 mm deep. Guide cannula was secured to the skull by application of cyanoacrylate cement (Scotch Super Glue) on the underside of the cannula and sealed by dental cement. The procedure was performed using 2% (vol/vol) isofluorane inhalation anesthesia on an isothermal pad at 37 °C. Buprenorphine and carprofen were administered post-operatively to reduce pain and discomfort. Mice were allowed to recover fully before being returned to cages and a hydrogel water source and food pellets were placed on the floor of the cage to prevent dislodgment of cannulas by snagging during the treatment period.

For the actute dose response study 2 month old *Cln1*$^{-/-}$ mice implanted with a guide cannula received an ICV infusion of either 20 μg or 40 μg (5 μl or 10 μl of the same rhPPT1 preparation used throughout this study), or vehicle (n=3 per treatment group). 24 hours later mice were sacrificed, together with n=3 age-matched naïve untreated wild type mice and the specific PPT1 enzyme activity was calculated in the infused hemisphere of each mouse and corrected per nanogram of protein (as described below).

For monthly infusions, mice were anesthetized using vaporizer settings of 2% (vol/vol) isoflurane anesthesia and placed an isothermal pad at 37 °C. ICV-treated mice received 5 μl of rhPPT1 infusion with the internal injector was connected via cannula tubing (PE50, inner diameter 0.58 mm and outer diameter 1.27 mm) to a Harvard syringe pump (Harvard Apparatus) at a rate of 1 μl/min. IT-treated mice received a bolus dose of the same volume into the intra-vertebral space between L4 and L5 as previously described using a 0.5 ml

syringe and 30 gauge needle (5). Combined ICV and IT (ICV+IT) treated mice received a split dose (2.5µl) via each route.

rhPPT1 administration in Sheep

Single dose

6-month-old *CLN1*^{R151X} sheep (n=4) received a 0.5ml single dose of rhPPT1 into the left lateral ventricle of the brain using a Hamilton syringe following guidance into place by an expert neuroradiologist with CT and fluoroscopy and location confirmed with injection of 0.3-0.5ml of gadopentetate dimeglumine (0.5 mmol/mL; Optiray (Ioversol) 350 injection, Guerbet LLC, Princeton, NJ) contrast agent.

Repeated dosing

Under general anesthesia 6-month-old *CLN1*^{R151X} sheep (n=2) were implanted with an intraventricular catheter into the left lateral ventricle with a guide cannula using CT and fluoroscopy directed surgery guided by an expert neuroradiologist. Catheters had a sub-dermal injection port for subsequent infusions (Solomon mid lovol port, Cat no MIDLOA-C50, Access technologies, Illinois, USA; Catheter ICV 3F Tubing, 847 356 0321, SAI Infusion Technologies; Norfolk medical "huber point" needle 22Ga x ¾" with transparent hub, Cat NMP-HN-22-75, Access technologies, Illinois, USA) fixed into place with 3 screws on into the skull at the site of port placement, adjacent to catheter entry into the skull. Enzyme was infused into port with 36" Ultra-Microbore extension set with side clamp and male/female Luer locks, cat no EXT-36 SAI infusion technologies, Illinois, USA). Injection site was confirmed using C-Arm. Prior to monthly infusions, sheep were anesthetized and the patency of the catheter was confirmed by injection of contrast agent (gadopentetate

dimeglumine (0.5 mmol/mL; Optiray (Ioversol) 350 injection, Guerbet LLC) which was confirmed by computerized tomography (CT) scanning or fluoroscopy. Sheep received monthly infusions of the same batch of rhPPT1 as used in the mice at 4 mg/ml concentration at an appropriately scaled dose of 1 ml of rhPPT1 (~200x dose used for mice) at a rate of 0.6 ml/hour infusion. Post-surgery and/or administration of rhPPT1, the animals were monitored continuously during the recovery period. The sheep were fully recovered and ambulatory before being returned to their accommodation in individual pens. Treated sheep were monitored for at least one week post any surgical procedure and treated with analgesics when necessary.

Pre- and post-operative anesthesia and care of sheep

Sheep were sedated with medetomidine (5 μgkg^{-1}; "Medetor"; Virbac, Suffolk, UK), ketamine (0.5 mg/kg; "Ketamidor"; Chanelle Vet (UK), Berkshire, UK), combined and administered by slow injection into the jugular vein. When profound sedation was present (2-18 minutes), anesthesia was induced with propofol ("Propofol 1% w/v", Fresenius Kabi, Cheshire, UK) administered to effect (2-5 mg/kg) by slow injection into the jugular vein. Once the sheep was unconscious, its trachea was intubated with an endotracheal tube (7.5-10 mm internal diameter) and the cuff inflated.

The sheep remained in sternal recumbency throughout anesthesia, which was maintained using isoflurane ("IsoFlo"; Zoetis, Surrey) vaporized in oxygen and medical air (1:1), administered via a 'circle' rebreathing system. Mechanical ventilation was imposed using a Datex Aestiva 5 anesthesia workstation (Datex Ohmeda, Helsinki, Finland) in volume-control mode, with a tidal volume of 7-11 ml/kg and positive end expiratory pressure of 2-4

cmH_2O. Respiratory rate and/or tidal volume were adjusted to avoid excessive hypercapnia (Fe'CO_2 4.7-7.3 kPa).

Physiological monitoring during anesthesia consisted of electrocardiogram, pulse oximetry, inspired and expired concentrations of O_2, CO_2 and inhalant agent, spirometry, temperature (intranasal or rectal) ("S/5 Compact"; Datex Ohmeda, Helsinki, Finland). Blood pressure was monitored invasively, using an arterial cannula (20G or 22G) placed in the central auricular artery, or non-invasively, using an appropriately sized cuff placed around a distal limb. Intravenous cannulation of the jugular (16G) or marginal auricular (20G) vein was undertaken to allow administration of Hartmann's solution ("Vetivex No11"; Dechra, Shrewsbury) at 2-5 mL/kg/hr.

For surgical procedures, analgesia was provided using intravenous buprenorphine (5 µg/kg "Buprecare", Animalcare, Yorkshire, UK) and either flunixin (2.2 mg/kg intravenously; "Finadyne", MSD Animal Health, Buckinghamshire, UK) or meloxicam (0.5 mg/kg subcutaneously; "Metacam", Boehringer Ingelheim Animal Health, Berkshire, UK). Peri-operative antibiosis was provided by intramuscular amoxicillin (15 mg/kg "Betamox LA, Norbrook Laboratories, Northamptonshire, UK). Two sheep also received dexamethasone (0.3 mg/kg intravenously; "Dexadreson", MSD Animal Health, Buckinghamshire, UK) at the time of port placement. Two sheep required needle centesis of the rumen to treat tympany (14G x 2" needle).

On completion of the procedure, isoflurane administration was ended and the sheep were weaned from the ventilator. The trachea was extubated once the sheep were breathing spontaneously and when active chewing and/or swallowing was present. The endotracheal

tube was withdrawn with the cuff partially inflated in order to remove any accumulated oropharyngeo-tracheal fluid. After this the intravenous cannula was removed. The sheep were monitored continuously until standing. One sheep received atipamezole (25 μg/kg intramuscularly; "Antisedan", Vetoquinol, Buckinghamshire) to hasten recovery.

Motor performance analyses in mice

Beginning at 1 month, mice were tested on the Rotarod (Rotamex 5, Columbus instruments, OH). For the stationary paradigm, mice were placed on mouse spindles (3 cm x 9.5 cm) for a maximum of 60 seconds. For the constant speed paradigm, mice were placed on spindle with a rotation speed of 2.5 RPM for a maximum of 60 seconds as before (6). Gait analysis was performed using the *CatWalk XT* (Noldus Information Technology bv, Wagenigen, Netherlands) semi-automated gait analysis system, as before (7). Briefly, mice were trained at least 2 days prior to data analysis and habituated to the room where behavior was performed overnight before testing. Each mouse had run durations ranging from 1 to 5 seconds to be considered compliant. *Catwalk XT* 10.5 software (Noldus Information Technology) was then used to analyze different parameters associated with gait for each individual paw - Right Fore (RF), Left Fore (LF), Right Hind (RH) or Left Hind (LH), in addition to removing any unwanted traces due to other parts of the body (e.g. mouse's belly, scrotum or tail). Data for individual limbs were then averaged for each run for individual animals where average data are shown. These data were then exported into an MS Excel spreadsheet (Microsoft, Seattle).

MRI analysis in sheep

Sheep were euthanized by the administration of intravenous pentobarbital (40 mg/kg) before undergoing imaging studies within 1 hour. MRI performed on a Siemens Skyra 3 T platform using a 4-channel flex coil wrapped tightly across the head. T1 weighted volumetric imaging at 0.8 mm isotropic resolution for structural assessments was acquired as part of a protocol which includes the MRI sequences which would be undertaken clinically. Since it is possible through structural imaging to differentiate homozygous mutant from wild-type and heterozygous animals (**Figure 5B**). T1w 3D images were co-registered to a whole head template image (8) using a 2-step process comprising linear affine registration initiated by moments of inertia followed by non-linear asymmetric diffeomorphic registration using the Greedy algorithm. Applying the inverse warp to an intracranial mask in template space allowed for atlas-based brain extraction in preference to poorly performing current native space algorithms which are not currently optimised for large animal livestock brains.

The extracted brains were subject to tissue classification using the ANTs (advanced normalisation tools) Atropos pipeline comprising N4 bias field correction followed by multivariate (T1w and T2w) Markov random field expectation maximisation segmentation for 3 tissue classes (cerebrospinal fluid (CSF), grey matter (GM), and white matter (WM)). This produces separate tissue probability maps for each class. A hard 3 class segmentation was derived from the 3 posterior tissue probability maps, and the GM and WM tissue probability maps were used to calculate cortical thickness using the Diffeomorphic Registration-based Cortical Thickness (DiReCT) algorithm (9). The extracted brains were then co-registered to the INRA brain only template using an adaptation of the same registration pipeline (10). The INRA multicomponent cortical and subcortical atlases were inverse warped using label interpolation to provide native space regional segmentation for

each animal. Bilateral structures were left as combined regions of interest as per the current INRA atlas.

Using Matlab 2019a, several metrics were calculated for each animal and group, comprising 2 homozygous animals each in the enzyme replacement therapy (ERT) intervention group, untreated homozygous animals and 2 wild-type animals. For each animal, total intracranial volume was calculated from the intracranial mask. Total brain volume was calculated from a combination of the GM and WM tissue class probability maps, and total volume each of GM, WM, and CSF for comparison. The grey matter volumes of individual cortical region segmentations were calculated from the product of each region and the cortical grey matter posterior probability. All metrics were adjusted for total intracranial volume (GM+WM+CSF), total brain volume (GM+WM), and total grey matter volume. While total intracranial volume is widely used in human studies to adjust for age and sex-related brain size variability, we have previously described that areas of cortical grey matter undergo differential degrees of atrophy in PPT1 mutant homozygous animals(11). Therefore, regional grey matter volumes were normalized to total grey matter volume to assess for relative preservation of cortical regions.

Tissue Collection

At 6 months of age, all mice for analysis were sacrificed 24 hours after their last infusion of rhPPT1 or vehicle. Mice were anesthetized using 2% isoflurane anesthesia and transcardially perfused with PBS. The brain and spinal column were dissected out. The brain was bisected in the sagittal plane and spinal column divided into cervical, thoracic and lumbosacral segments. One brain hemisphere and the thoracic segment of cord were frozen and stored at -80°C for biochemical analysis, while the other brain hemisphere and cervical and lumbo-

sacral segments of cord were fixed in 4% paraformaldehyde (PFA) solution for 48 hours and subsequently cryoprotected in 30% sucrose in 50 mM TBS (pH=7.6).

At 6 months of age, sheep which received a single dose of rhPPT1, and their age-matched controls, were euthanized using intravenous pentobarbital (40 mg/kg) 24 hours, 1 week, 2 weeks and 1 month post dosing. Blood and CSF were collected and stored at -80°C. The brain, spinal cord, liver, heart, spleen, kidneys and eyes were dissected out. The left hemisphere of the brain, which contained the injection site, was stored at -80°C for biochemical analysis and the right hemisphere was immersed into 10% buffered formalin for fixation for 7-10 days prior to storage in 50 mM tris, 0.05% azide buffer (pH7.6) at 4°C. Cervical, thoracic and lumbar spinal cord sections, and all other resected samples, were divided for subsequent biochemical and histopathological examinations as described for the brain. Sheep that received repeated dosing were euthanized at 13 months of age after receiving rhPPT1 on a monthly basis. After removal of the ports the sampling procedure at autopsy was identical to those sheep that received single dose of enzyme.

PPT1 Assay of sheep tissues

CSF was collected into EDTA coated vacutainers from the lumbar region of the sheep immediately after euthanasia. The 1 step PPT1 assay protocol was employed as described in 14. Briefly, 20 ul undiluted CSF was added to 20ul of substrate solution (0.64 mM MU-6S-Palm ß Glc, 15 mM dithiothreitol (DTT), 0.375% (w/v) Triton X-100, and 0.1 ß glucosidase from almonds (Sigma) in McIlvain's phosphate/ citrate buffer (pH 4.0). The reaction was incubated overnight (18–24 hours) at 37 °C and terminated by addition of 0.5 M NaCO3/NaHCO3 (pH 10.7) containing 0.025% TritonX-100. Samples were read on a fluorometer at 405 nm (360 nm excitation and 460 nm emission).

Brain punches were sampled from 5 areas in the plane of the injection site/enzyme delivery area; Site 1- subventricular area, Site 2 - cortex above the ventricle, Site 3 - the cortical gyri (where injection site could be located), Site 4 - striatum/thalamic are and Site 5 - brain stem. Tissues collected from the 5 sites from each animal were homogenized with a hand-held homogenizer in distilled water prior to sonication using a Bioruptor Pico sonication device (Diagenode, Belgium). Samples were then centrifuged at 2000xg for 10 minutes at 4°C. The supernatant portion of the homogenate was used for the assay with the protein concentration determined using micro BCA assay (Life technologies, UK). 10 µl of homogenate containing 5 ug of protein was added to 20 µl of substrate solution (0.64 mM MU-6S-Palm ß Glc, 15 mM dithiothreitol (DTT), 0.375% (w/v) Triton X-100, and 0.1 ß glucosidase from almonds (Sigma) in McIlvain's phosphate/ citrate buffer (pH 4.0). The reaction was incubated for 1 hour at 37 °C and terminated by addition of 0.5 M NaCO3/NaHCO3 (pH 10.7) containing 0.025% TritonX-100. Samples were read on a fluorometer at 405 nm (360 nm excitation and 460 nm emission).

Histopathological analyses

4% PFA-fixed and cryoprotected mouse brain and spinal cord tissue were sectioned along the coronal axis at 40µm using a Microm HM430 freezing microtome (Microm International, Germany) equipped with a with Physitemp BFS-40MPA freezing stage (Physitemp, Clifton, NJ). Formalin-fixed and cryoprotected sheep brain hemispheres were further divided into blocks beginning at the level of the ICV cannula rostrally and extending to the caudal extent of the thalamus. 50 µm coronal sections were cut on the same freezing microtome used for mouse tissues.

Immunostaining

A 1 in 6 series of brain tissue and a 1 in 48 series of spinal cord sections were mounted on to Colorfrost plus (Thermo Fisher Scientific) slides and air-dried for 45 mins. Sections were stained using a modified immunofluorescence protocol, as previously described (7). Briefly, sections were blocked in 15% goat serum solution (S-1000, Vector Laboratories, CA) in 2% TBS-T (1x Tris Buffered Saline, pH 7.6 with 4% Triton-X100, Fisher Scientific) for 1 hour followed by incubation in primary antibody in 10% serum solution in 4% TBS-T for 2 hours. Primaries used were as follows: Rabbit anti-Glial Fibrillary Acidic Protein (GFAP, Agilent, Z0334, 1:1000), Rat anti-CD68 (Bio-Rad, MCA1957, 1:400), Rabbit anti-ATP Synthase C (SCMAS) (Abcam, ab181243, 1:200). Slides were washed three times in TBS and incubated in Alexa-Fluor labelled IgG secondary antibodies (Alexa-Fluor goat anti-rabbit 488, A-11008, 1:500 and goat anti-rat 546, A-11081, 1:500, Invitrogen) in 10% serum solution in 4% TBS-T for 2 hours, washed three times and incubated in a 1x solution of TrueBlack lipofuscin autofluoresence quencher (Biotium, Fremont, CA) in 70% ethanol for 2 mins before rinsing in 1xTBS. Slides were coverslipped in Fluoromount-G mounting medium containing DAPI (Southern Biotech, Birmingham, AL).

Sheep brain sections were stained using a free-floating immunofluorescence protocol. Sections were placed in a 6-well plate (Fisher) and blocked in 15% goat serum solution (S-1000, Vector Laboratories) in 0.3% TBS-T for 1 hour followed by overnight incubation in primary antibodies in 10% goat serum in 0.3% TBS-T at 4˚C. Primary antibodies used were - Mouse anti-GFAP (G3893, Sigma, 1:200) and Rabbit anti-Iba1 (019-19741, FUJIFILM Wako, 1:200). Sections were washed 3x in TBS, and incubated in Alexa-Fluor secondaries (Alexa-Fluor goat anti-rabbit 546, A-11001, 1:500 and goat anti-mouse 488, A-11081,1:500, Invitrogen) in 10% goat serum in 0.3% TBS-T for 2 hours. Sections were then mounted on to Colorfrost plus (Thermo Fisher Scientific) slides and dried briefly before incubation in a 1x

solution of TrueBlack lipofuscin autofluoresence quencher (Biotium) in 70% ethanol for 2 mins and rinsing in 1xTBS. Slides were coverslipped in Fluoromount-G mounting medium containing DAPI (Southern Biotech). For visualisation of autofluorescent storage material (AFSM), sections were directly mounted on to Colorfrost plus (Thermo Fisher Scientific) slides, briefly air-dried and coverslipped with Fluoromount-G (Southern Biotech).

Cresyl fast violet staining

40 µm coronal mouse sections and 50 µm sheep sections were mounted were stained for cresyl fast violet (Nissl) to reveal their cytoarchitecture. For mouse tissue, a 1 in 6 series of brain and 1 in 48 series of spinal cord sections, while representative sheep brain sections at the level of the ICV cannula rostrally and thalamus caudally were mounted on chrome-gelatin coated microscope slides (ThermoFisher Scientific) and air-dried overnight. All sections were then incubated for at least 45 minutes at 60°C in 0.1% cresyl fast violet and 0.05% acetic acid. Stained sections were then differentiated through a series of graded ethanol solutions (70%, 80%, 90%, 95% and 2x100%) (Fisher Scientific, MA) before clearing in Xylene (Fisher Scientific) and coverslipping with DPX (Fisher Scientific), a xylene-based mountant.

Thresholding Image Analysis

To analyze the degree of storage material accumulation (SCMAS or AFSM) and glial activation in the gray matter (GFAP-positive astrocytes and CD68 or Iba-1-positive microglia), a semiautomated thresholding image analysis was used (7) with Image-Pro Premier software (Media Cybernetics, MD). Briefly, slide-scanned images at 10x magnification for stained sections were collected for all animals followed by demarcation of anatomical regions of interest. Images were subsequently analyzed using Image-Pro Premier (Media Cybernetics) using appropriate thresholds that selected the foreground

immunoreactivity above background. Separate thresholds were used for each antigen and each anatomical region analyzed.

Cortical Thickness & Neuron Counts

Cortical thickness was measured across the primary somatosensory barrel field (S1BF) (12) in mice and the primary somatosensory cortex in sheep (https://brains.anatomy.msu.edu/brains/sheep/) using *StereoInvestigator* software (MBF Bioscience,VT). This was done by drawing 10 individual contours over the entire thickness of cortex across 3 separate sections and collecting the average value in µm. Counts of neurons in the S1BF, ventral postero-medial and postero-lateral thalamic nucleus (VPM/VPL) and ventral horns of the spinal cord (SC VH) were performed by a design-based optical fractionator method in a one in six and forty-eight series of Cresyl fast violet-stained sections in the mouse brain and cord respectively using *StereoInvestigator* software (MBF Bioscience) (7, 13). Cells were sampled with counting frames (100 × 90µm) distributed over a sampling grid (S1BF 450x450 µm, VPM/VPL 350x350 µm, SC VH 300×300 µm) that was superimposed over the region of interest at 100× magnification. The Gundersen Co-efficient of error (m=1) was maintained between 0.05 and 0.1 (14, 15). Estimated neuron counts were then exported to MS Excel (Microsoft).

References

1. Gupta P, Soyombo AA, Atashband A, Wisniewski KE, Shelton JM, Richardson JA, et al. Disruption of PPT1 or PPT2 causes neuronal ceroid lipofuscinosis in knockout mice. *Proceedings of the National Academy of Sciences of the United States of America.* 2001;98(24):13566-71.
2. Eaton SL, and Wishart TM. Bridging the gap: large animal models in neurodegenerative research. *Mammalian Genome.* 2017;28(7-8):324-37.
3. Lu J-Y, Hu J, and Hofmann SL. Human recombinant palmitoyl-protein thioesterase-1 (PPT1) for preclinical evaluation of enzyme replacement therapy for infantile neuronal ceroid lipofuscinosis. *Molecular Genetics and Metabolism.* 2010;99(4):374-8.
4. Kan SH, Elsharkawi I, Le SQ, Prill H, Mangini L, Cooper JD, et al. Biochemical evaluation of intracerebroventricular rhNAGLU-IGF2 enzyme replacement therapy in neonatal mice with Sanfilippo B syndrome. *Mol Genet Metab.* 2021;133(2):185-92.
5. Shyng C, Nelvagal HR, Dearborn JT, Tyynela J, Schmidt RE, Sands MS, et al. Synergistic effects of treating the spinal cord and brain in CLN1 disease. *Proc Natl Acad Sci U S A.* 2017;114(29):E5920-E9.
6. Macauley SL, Wozniak DF, Kielar C, Tan Y, Cooper JD, and Sands MS. Cerebellar pathology and motor deficits in the palmitoyl protein thioesterase 1-deficient mouse. *Experimental Neurology.* 2009;217(1):124-35.
7. Nelvagal HR, Dearborn JT, Ostergaard JR, Sands MS, and Cooper JD. Spinal manifestations of CLN1 disease start during the early postnatal period. *Neuropathol Appl Neurobiol.* 2021;47(2):251-67.
8. Das SR, Avants BB, Grossman M, and Gee JC. Registration based cortical thickness measurement. *Neuroimage.* 2009;45(3):867-79.

9. Nitzsche B, Frey S, Collins LD, Seeger J, Lobsien D, Dreyer A, et al. A stereotaxic, population-averaged T1w ovine brain atlas including cerebral morphology and tissue volumes. *Front Neuroanat.* 2015;9:69.

10. Ella A, Delgadillo JA, Chemineau P, and Keller M. Computation of a high-resolution MRI 3D stereotaxic atlas of the sheep brain. *J Comp Neurol.* 2017;525(3):676-92.

11. Eaton SL, Proudfoot C, Lillico SG, Skehel P, Kline RA, Hamer K, et al. CRISPR/Cas9 mediated generation of an ovine model for infantile neuronal ceroid lipofuscinosis (CLN1 disease). *Scientific Reports.* 2019;9(1):9891-.

12. Paxinos Franklin KBJG. *The Mouse Brain in Stereotaxic Coordinates, 2nd ed.* Academic Press, San Diego.; 2001.

13. Kielar C, Maddox L, Bible E, Pontikis CC, Macauley SL, Griffey MA, et al. Successive neuron loss in the thalamus and cortex in a mouse model of infantile neuronal ceroid lipofuscinosis. *Neurobiology of Disease.* 2007;25(1):150-62.

14. West MJ. Design-based stereological methods for counting neurons. *Prog Brain Res.* 2002;135:43-51.

15. Gundersen HJ, Jensen EB, Kiêu K, and Nielsen J. The efficiency of systematic sampling in stereology--reconsidered. *Journal of microscopy.* 1999;193(Pt 3):199-211.

Supplementary Figures

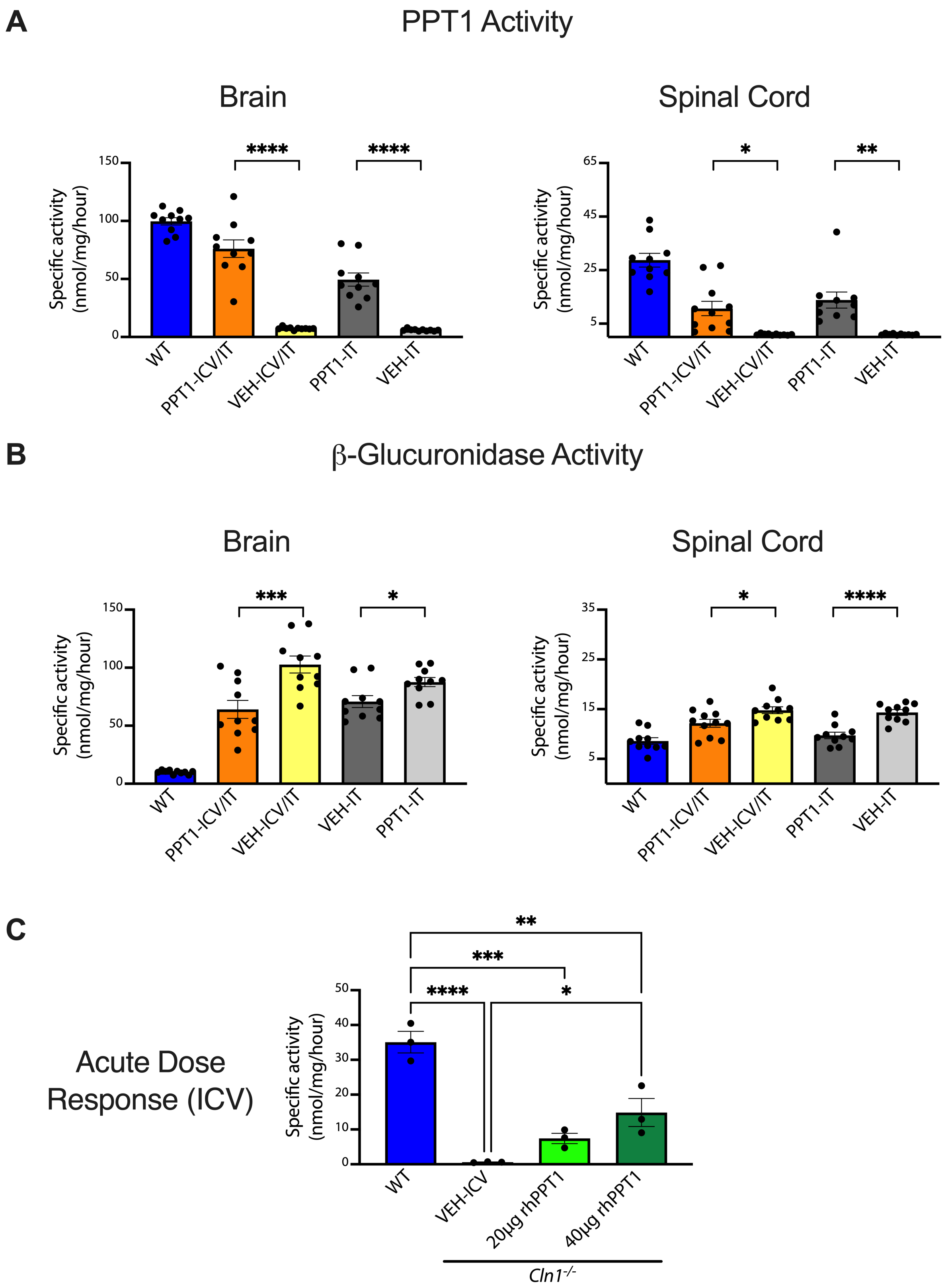

Figure S1 PPT1 and β-glucuronidase activity after different routes and doses of administration of rhPPT1 in Cln1$^{-/-}$ mice.

Specific activity in nmol/mg/hr of PPT1 (**A**) and β-glucuronidase (**B**) enzymes from homogenates of tissue collected from mice 24 hours after their last infusion via Intrathecal (IT) or combined intracerebroventricular and IT (ICV/IT) routes showing statistically significant increase in PPT1 activity and reduction in β-glucuronidase in both the brain and spinal cord of treated mice (PPT1-ICV/IT and PPT1-IT) as compared to vehicle treated controls (VEH-ICV/IT and VEH-IT). However, these enzyme values were not normalized to wild-type (WT) control values. (n=10). (**C**) Acute dose response study showing brain PPT1 enzyme specific activity in nmol/mg/hr following intracerebroventricular (ICV) administration of the rhPPT1 or vehicle to young adult (2 mo) *Cln1*$^{-/-}$ mice, compared to naïve uninfused wild type mice (WT) (n=3). Delivery of 40 µg rhPPT1 (twice the administered 20 µg dose used throughout this study) doubles the level of PPT1 enzyme activity present 24 hours after infusion. Note that the absolute specific activity in this acute study is not comparable to that seen in chronically treated mice (that received seven enzyme infusions in total, **Fig. 1D**, **Fig. S1A**) in which the low level of residual PPT1 activity that persists for several weeks accumulates after each infusion (16). Data represent mean ± SEM. One-Way ANOVA with *post-hoc* Bonferroni correction. n=10; *p<0.05, **p<0.01, ***p<0.001, ****p<0.0001 (See Data File S2 for full p values).

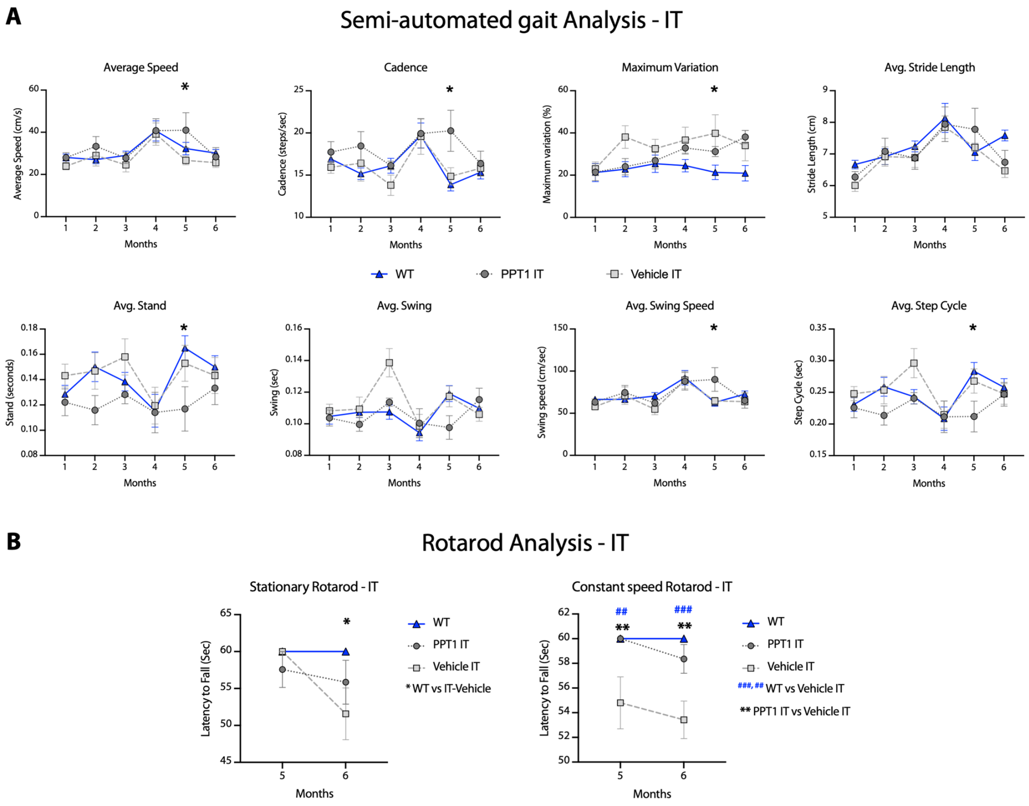

Figure S2 motor performance in IT treated *Cln1*$^{-/-}$ mice.

(**A**) Semi-automated gait analysis measures of average speed (cm/s), cadence (steps/second), maximum variation of speed (%), Stride length (cm), Stand (s), Swing (s), Swing speed (cm/s) and step cycle (s) from 1-6 months showing overall performance of intrathecal treated (PPT1-IT) mice compared to vehicle treated (Vehicle IT) and wild-type (WT) values. (**B**) Stationary and constant-speed rotarod tests in 5- and 6- month old mice. PPT1-IT mice showed a non-significant reduction in performance to WT while Vehicle IT mice showed a statistically significant reduction in latency to fall (s) in the stationary rotarod test at 6 months. Vehicle IT mice showed a reduced latency to fall at 5 and 6 months in constant speed rotarod test. Data represent mean ± SEM. Two-way ANOVA (mixed-effects) with *post-hoc* Bonferroni correction. n=10; *p<0.05, **p<0.01, ***p<0.001, ****p<0.0001 (See Data File S2 for full p values).

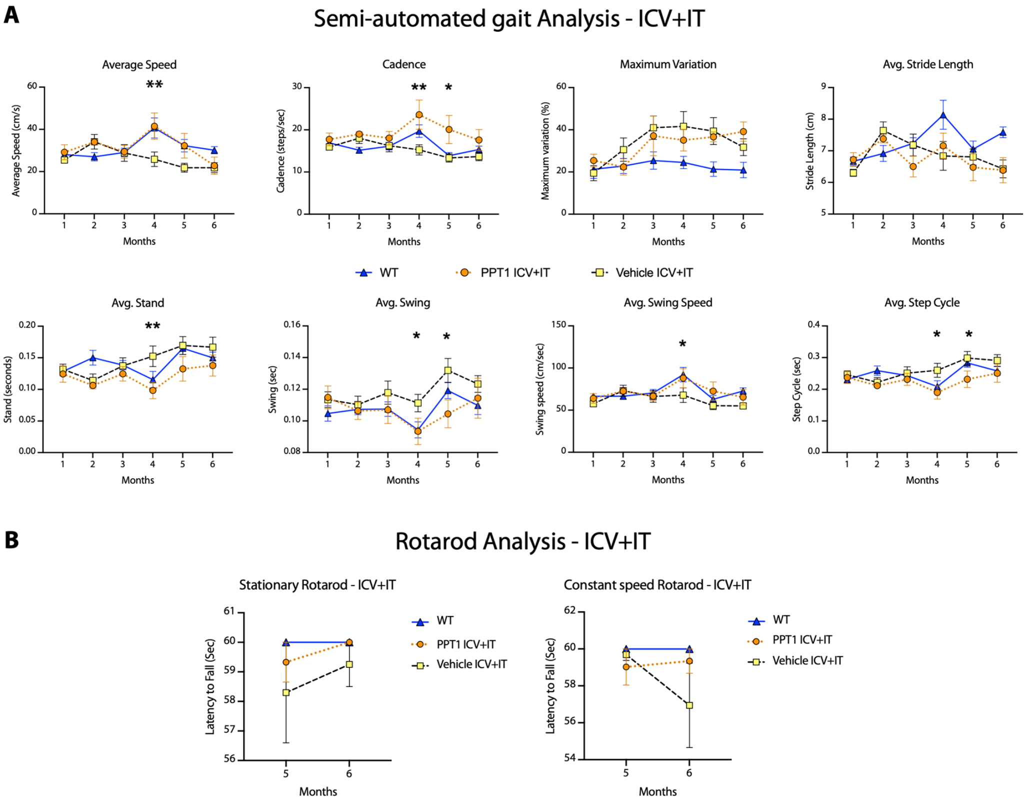

Figure S3 motor performance in ICV+IT combination treated *Cln1*$^{-/-}$ mice.

(**A**) Semi-automated gait analysis measures of average speed (cm/s), cadence (steps/second), maximum variation of speed (%), Stride length (cm), Stand (s), Swing (s), Swing speed (cm/s) and step cycle (s) from 1-6 months showing moderately improved performance of combined intracerebroventricular and intrathecal treated (PPT1-ICV+IT) mice compared to vehicle treated (Vehicle ICV+IT), but not normalized to wild-type (WT) values. (**B**) Stationary and constant-speed rotarod tests in 5- and 6- month old mice. PPT1-ICV+IT and Vehicle ICV+IT mice showed non-significant trends in performance to WT mice across both tests. Data represent mean ± SEM. Two-way ANOVA (mixed-effects) with *post-hoc* Bonferroni correction. n=10; *p<0.05, **p<0.01, ***p<0.001, ****p<0.0001 (See Data File S2 for full p values).

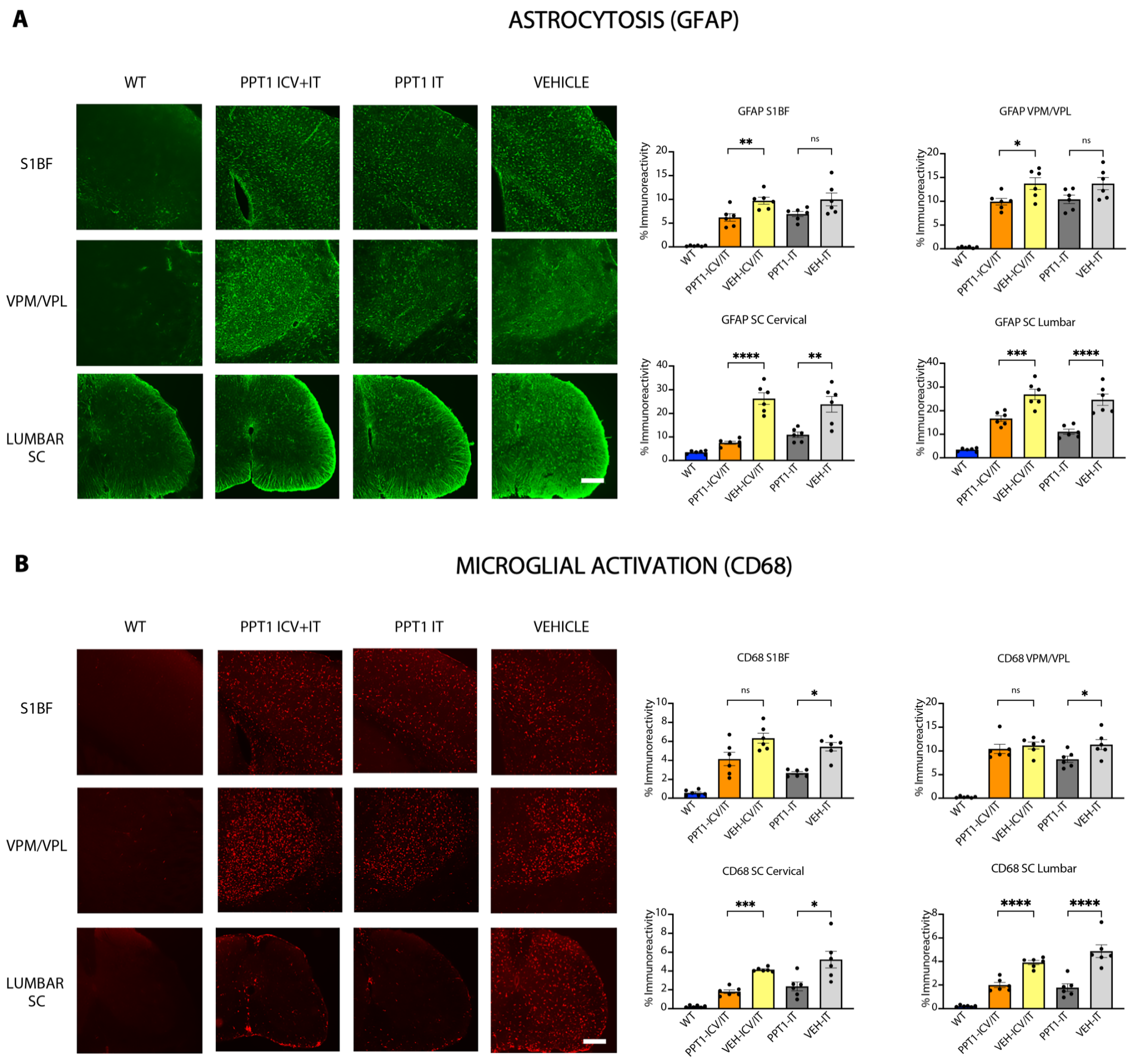

Figure S4. Decreased astrocytosis and microglial activation in brain and spinal cord of ICV+IT and IT treated *Cln1*$^{-/-}$ mice.

Representative 10x fluorescence images and thresholding image analysis histograms for (**A**) astrocytosis (GFAP) and (**B**) microglial activation (CD68) across the primary somatosensory cortex (S1BF), ventral-posterior thalamic nucleus (VPM/VPL) and cervical and lumbar spinal cord (SC). Intrathecal treated (PPT1-IT) mice showed statistically significant reduction of GFAP immunoreactivity in the spinal cord compared to Vehicle IT (VEH-IT) mice, but not in the brain. PPT1-IT mice showed a statistically significant reduction in CD68 immunoreactivity across all regions compared to VEH-IT mice. Conversely, combination intracerebroventricular and intrathecal (PPT1-ICV+IT) mice showed a statistically significant

reduction of GFAP positive astrocytes across all regions compared to Vehicle ICV+IT (VEH-ICV+IT) mice but only showed a significant reduction of CD68 positive microglia in the spinal cord and not the brain. Scale Bar = 100 µm. Data represent mean ± SEM. One-Way ANOVA with *post-hoc* Bonferroni correction. n=6; ns = not significant, *p<0.05, **p<0.01, ***p<0.001, ****p<0.0001 (See Data File S2 for full p values).

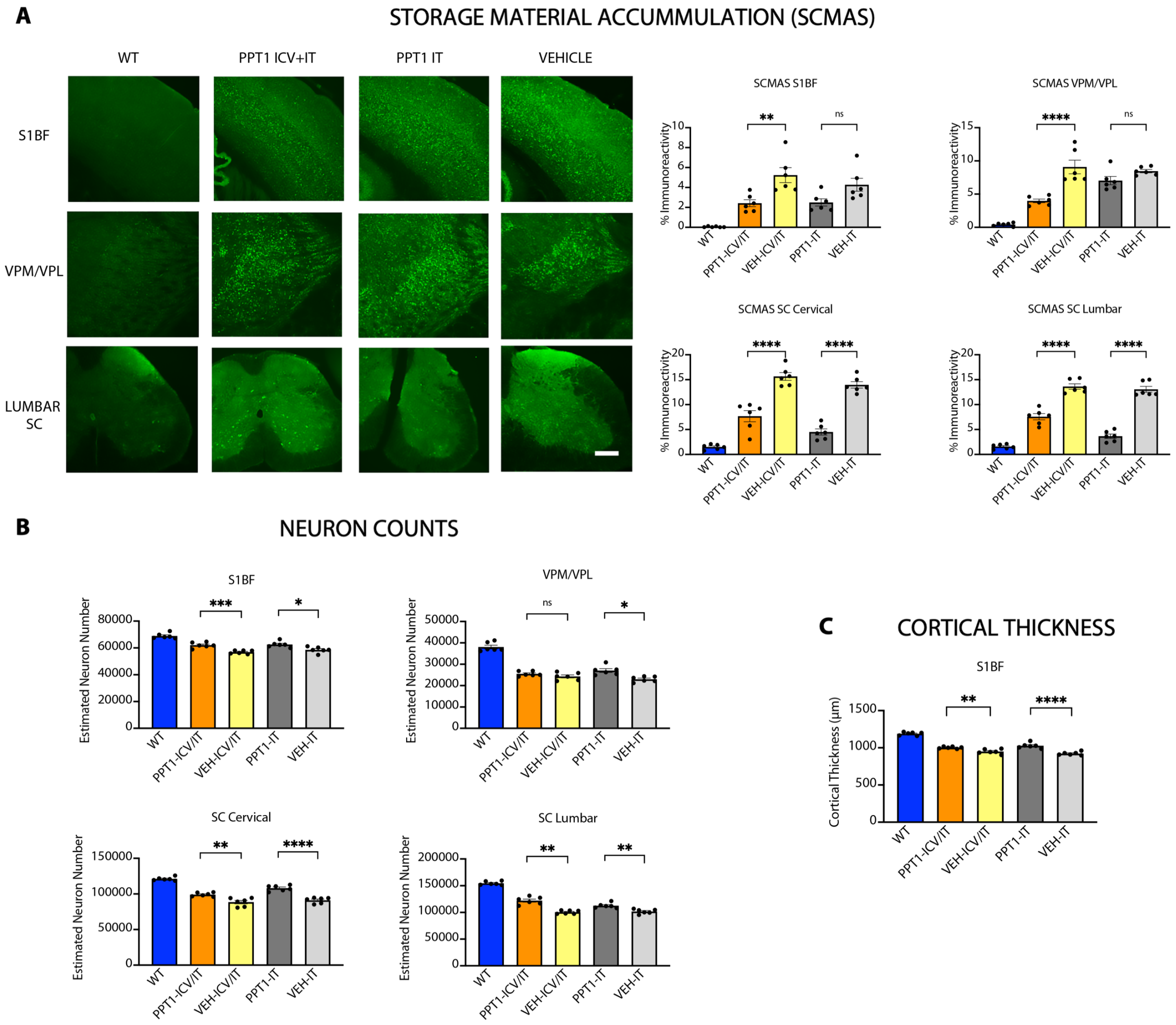

Figure S5. Storage material accumulation, neuron survival and cortical atrophy in ICV+IT and IT treated *Cln1*$^{-/-}$ mice.

(A) Representative 10x fluorescence images and thresholding image analysis for subunit C of mitochondrial ATPase (SCMAS) across the primary somatosensory cortex (S1BF), ventral-posterior thalamic nucleus (VPM/VPL) and cervical and lumbar spinal cord (SC), showing an overall statistically significant reduction in combination intracerebroventricular and intrathecal (PPT1-ICV+IT) and intrathecal treated (PPT1-IT) mice compared to Vehicle treated (VEH-ICV+IT and VEH-IT) across all regions except for PPT1 IT treated mice in the VPM/VPL. Scale bar = 50 µm. (B) PPT1-ICV+IT and PPT1-IT mice showed a statistically significant improvement in neuron number across all regions as compared to vehicle treated

controls (VEH-ICV+IT and VEH-IT) except in the VPM/VPL of PPT1-ICV+IT mice, which was not statistically significant. (C) There was also a significant rescue of cortical atrophy in the S1BF in both PPT1-ICV+IT and PPT1-IT treated mice compared to vehicle treated controls. Data represent mean ± SEM. One-Way ANOVA with *post-hoc* Bonferroni correction. n=6; ns = not significant, *$p<0.05$, **$p<0.01$, ***$p<0.001$, ****$p<0.0001$ (See Data File S2 for full p values).

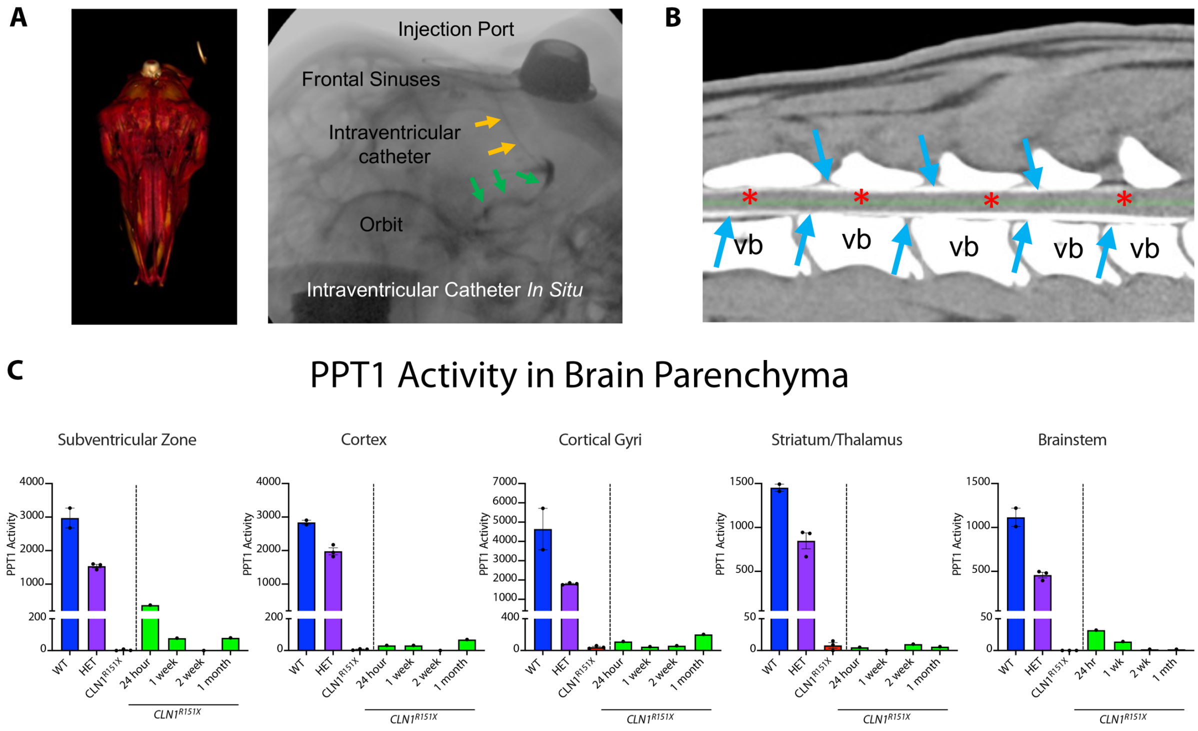

Figure S6. ICV infusion of rhPPT1 in CLN1^{R151X} sheep: Biodistribution to spinal cord and PPT1 activity across different brain regions.

(A) Representative CAT scan and MRI radiograph showing the placement of the injection port and transcranial intraventricular catheter (yellow arrows) for rhPPT1 infusions. Radio-dense contrast can be seen in the lateral ventricle (green arrows) confirming catheter patency and lateral ventricular tip location. **(B)** Reconstruction CT scan of the cervicothoracic spinal cord 75 minutes following a single intracerebroventricular (ICV) injection of contrast agent demonstrates presence of contrast agent in the intrathecal space (white signal, indicated by blue arrows) adjacent to the spinal cord (red *), and also extends to the lumbar cord (vb= vertebral body); indicating that ICV injected substances reach the spinal cord in sheep. **(C)** PPT1 activity (arbitrary units), measured in different regions of CLN1^{R151X} sheep (n=1) at 24 hr, 1 wk, 2 wk and 1 months after infusion showing slightly increased values compared to untreated CLN1^{R151X} sheep, but far less than that seen in with wild-type (WT) or

heterozygous (HET) sheep. Data represent mean ± SEM. (n=3 WT, 3 HET, 3 $CLN1^{R151X}$ and 1 for each treatment)

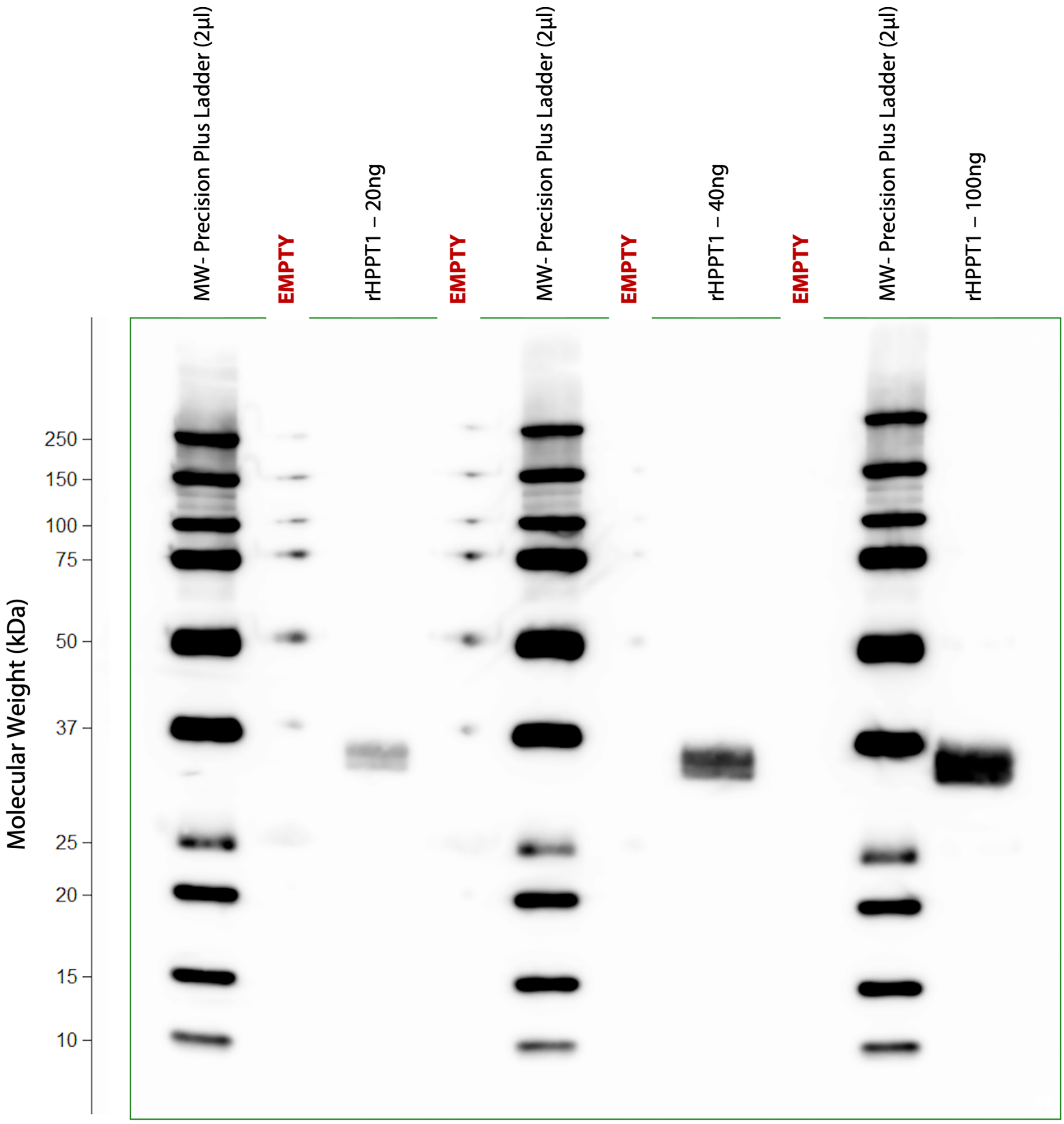

Figure S7. Western blot analysis of rhPPT1 from CHO cells

Raw western blot image of western blot stained for PPT1 after loading 20, 40 and 100ng of rhPPT1from CHO lysates with appropriate ladder showing a specific band of increasing intensity at just below 37kDa.

Region No.	Cortical Region	Pattern of Cortical Thickness
1	Anterior Sygmoideus Gyrus	Large treatment effect
3	Cingulate Gyrus	Moderate treatment effect
4	Claustrocortex (Insular Cortex)	No Apparent treatment effect
5	Ectolateralis Gyrus	Moderate treatment effect
6	Entolateralis Gyrus	Moderate treatment effect
7	Gyrus Rectus	Moderate treatment effect
8	Lateral Gyrus	Moderate treatment effect
11	Occipital Lobe	Moderate treatment effect
14	Orbital Gyrus	Moderate treatment effect
15	Orbitofrontal Gyrus	No Apparent treatment effect
16	Parahippocampal Cortex	No Apparent treatment effect
19	Postcruciate Gyrus*	Large treatment effect
20	Posterior Sygmoideus Gyrus	No Apparent treatment effect
21	Posterior Sylvian Gyrus	Moderate treatment effect
22	Precruciate Gyrus *	Large treatment effect
23	Suprasylvian Gyrus	Moderate treatment effect
24	Sylvian Gyrus	No Apparent treatment effect
25	Temporal Lobe	No Apparent treatment effect

Supplemental Table 1. Summary of histogram analysis of ovine cortical thickness

ANOVA was used for multiple comparison and treatment effects were classified based on the significance, order, and magnitude of differences between wild-type, ERT treated homozygous ($CLN1^{R151X}$ +rhPPT1), and untreated homozygous animals ($CLN1^{R151X}$). * Regions shown in Figure 5C roughly corresponding to primary motor (precruciate) and somatosensory (postcruciate) cortex respectively.
