# Supplementary material for: Cross-species efficacy of enzyme replacement therapy for CLN1 disease in mice and sheep
Source: J Clin Invest. 2022 Oct 17;132(20):e163107. doi: 10.1172/JCI163107 (PMC9566914; doi:10.1172/JCI163107)

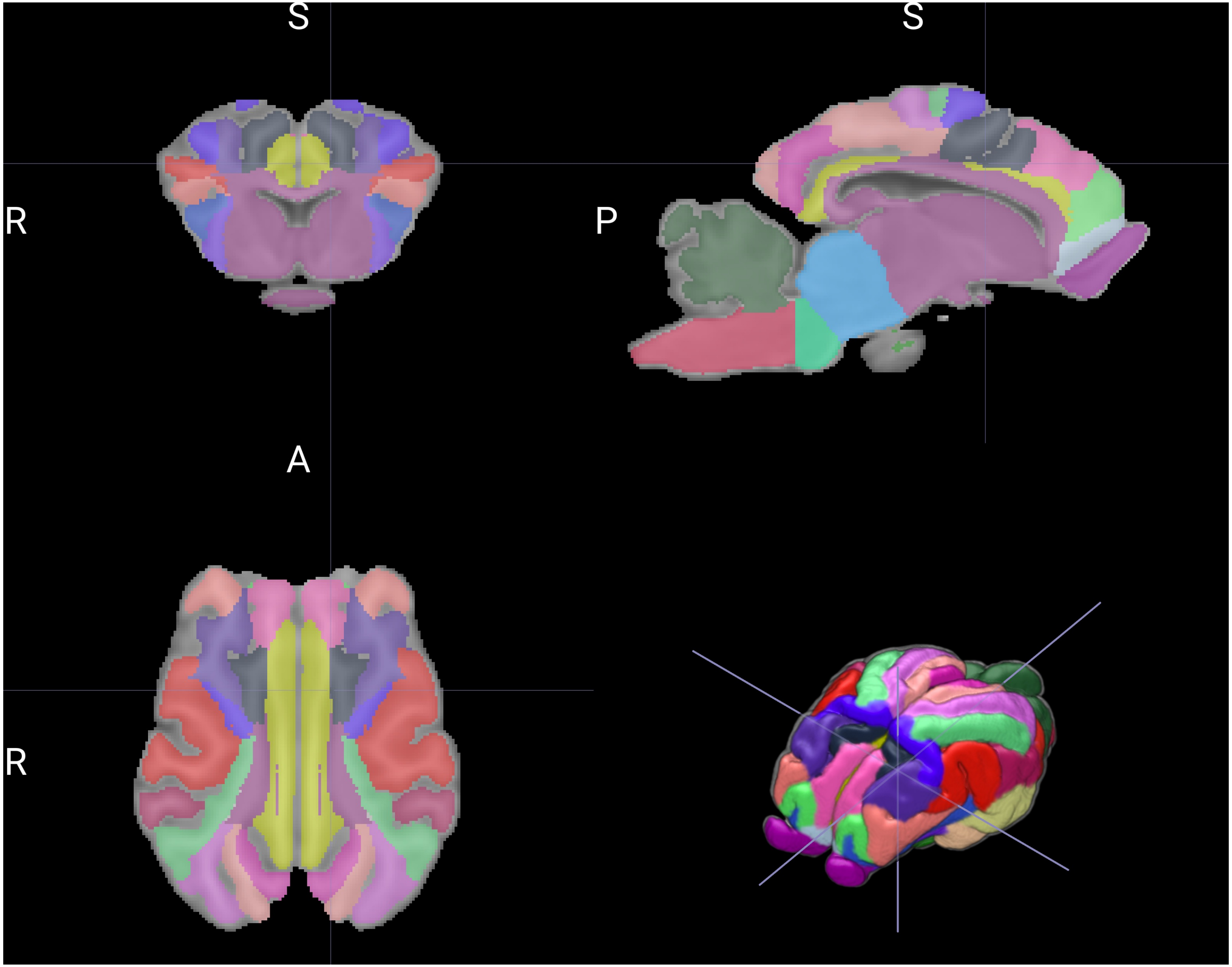

Schematic 3D represenatation of the different brain regions based on the INRA ovine atlas (*24*) used for analysis of cortical thicknesses (See following histograms)

Anterior Sygmoideus Gyrus

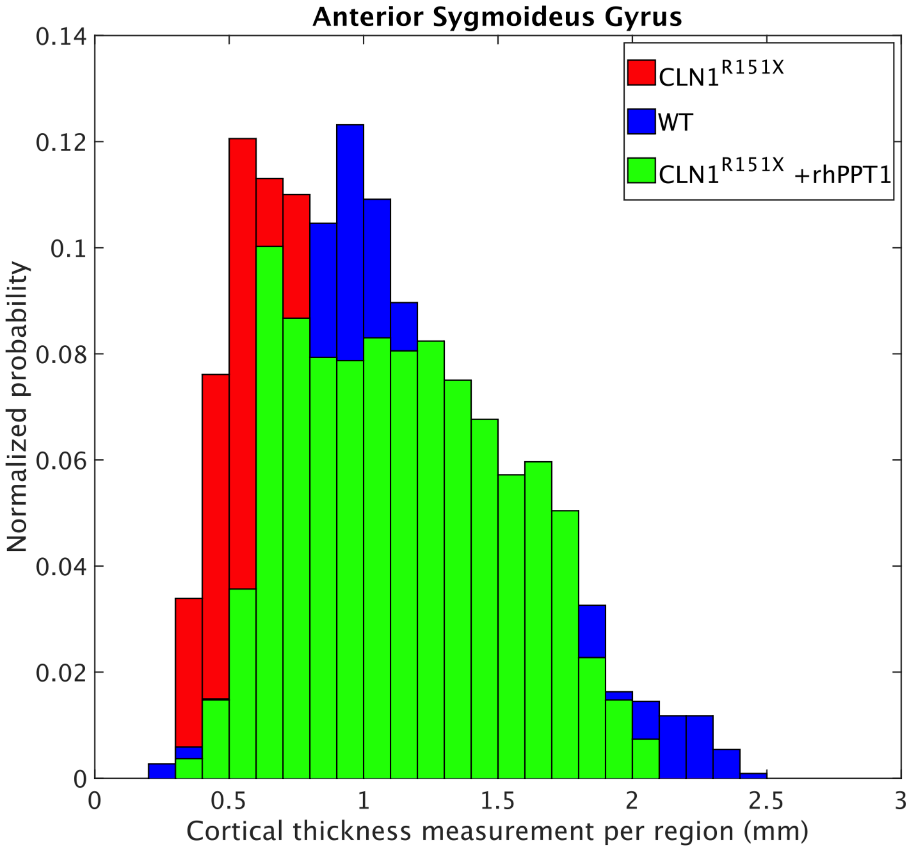

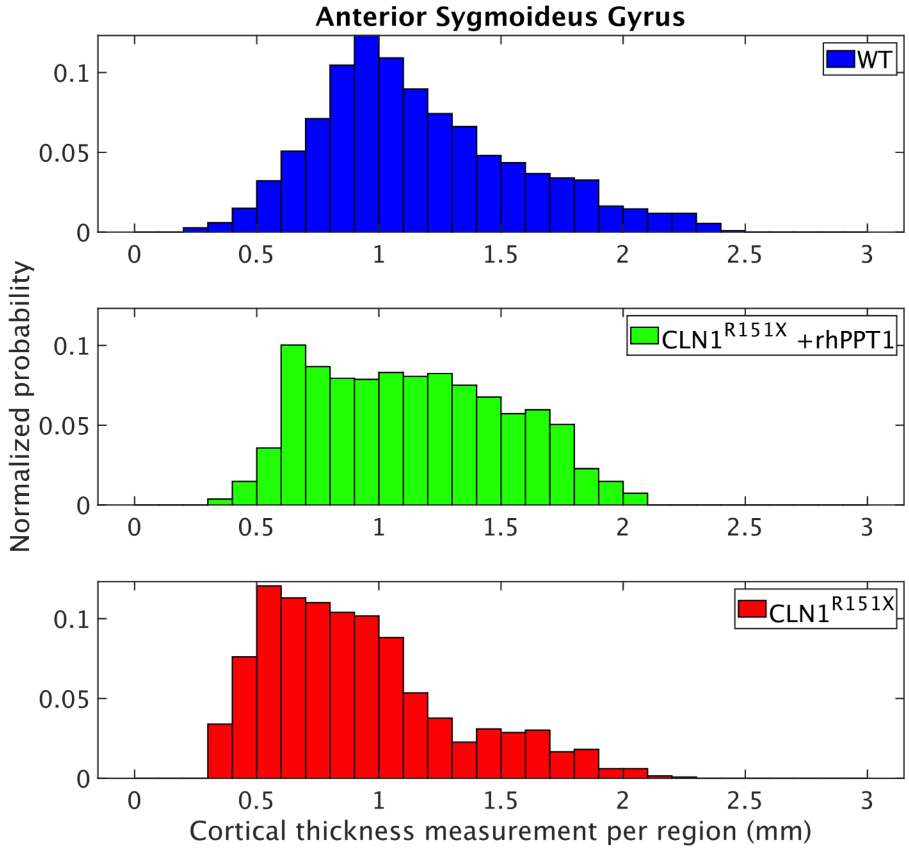

Cingulate Gyrus

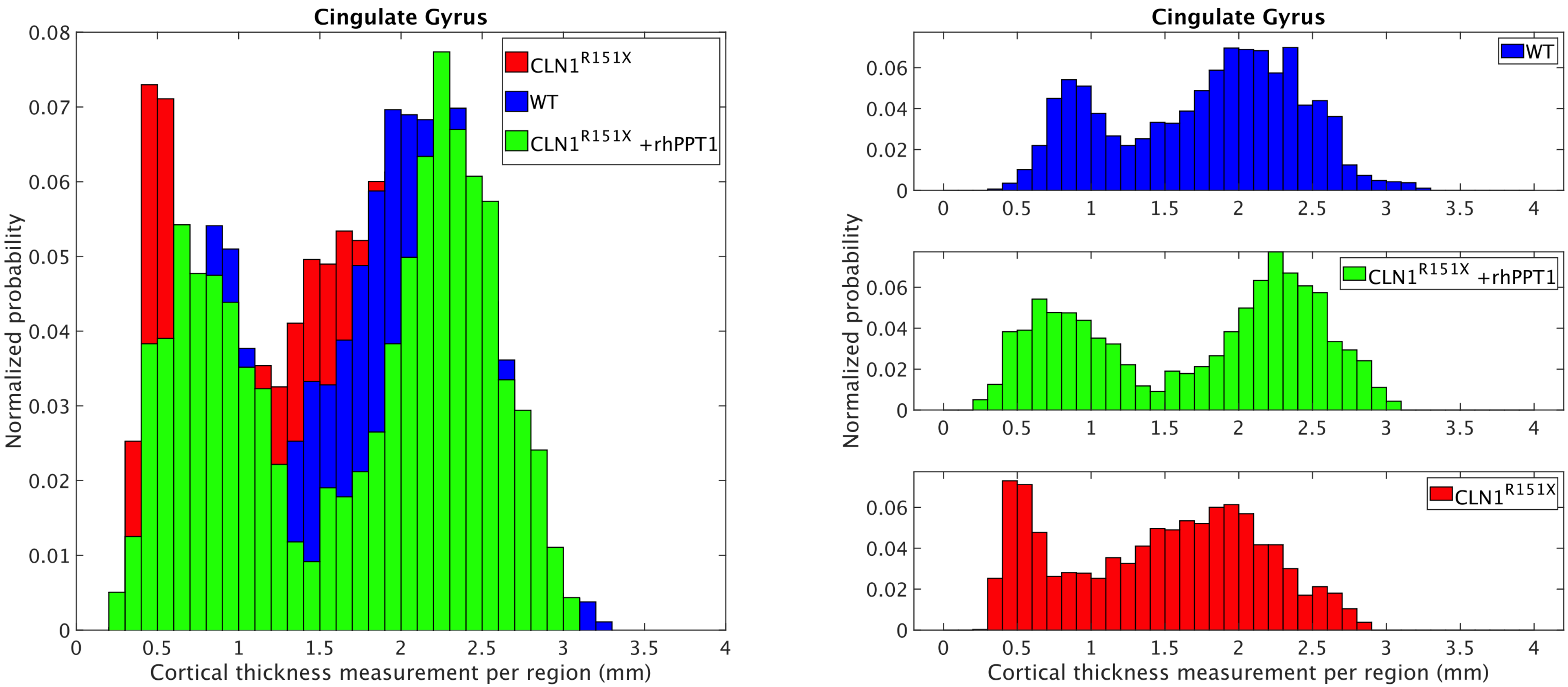

Claustrocortex (Insular Cortex)

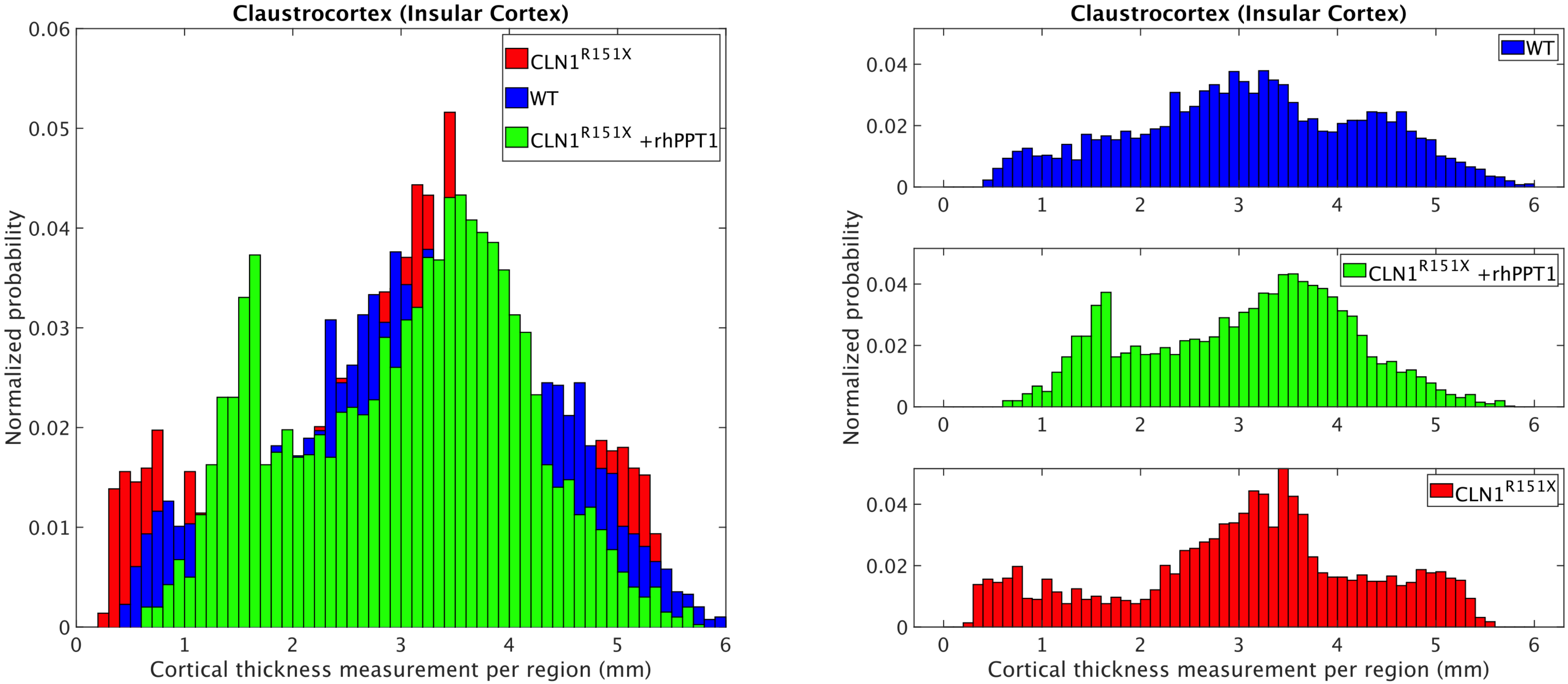

Ectolateralis Gyrus

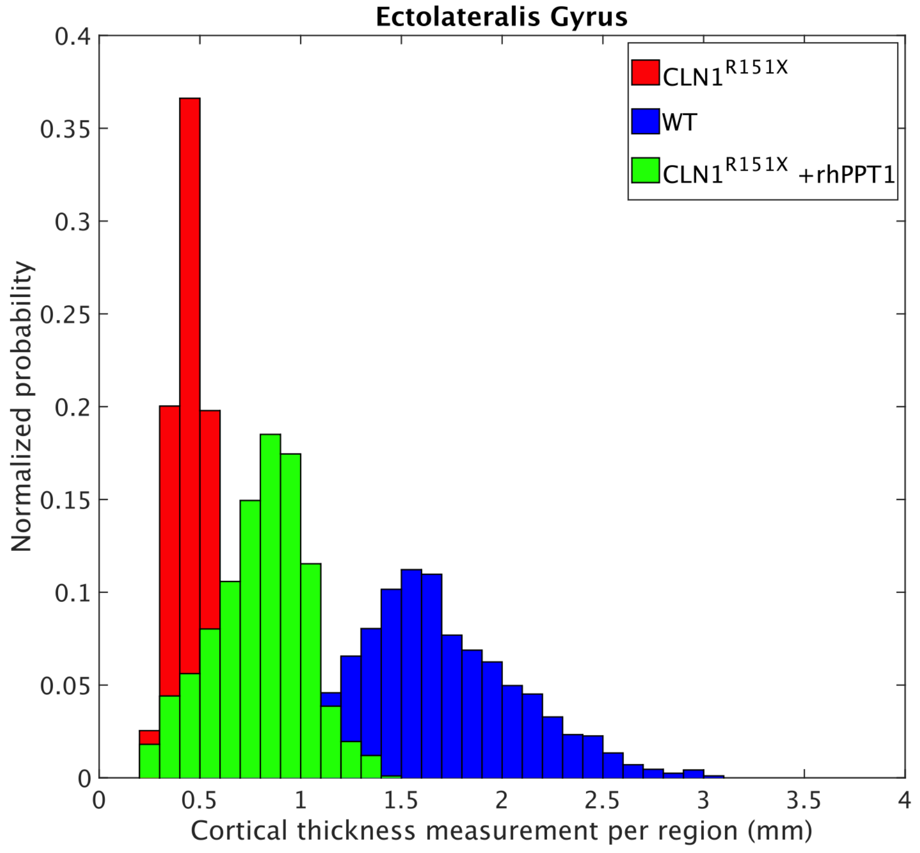

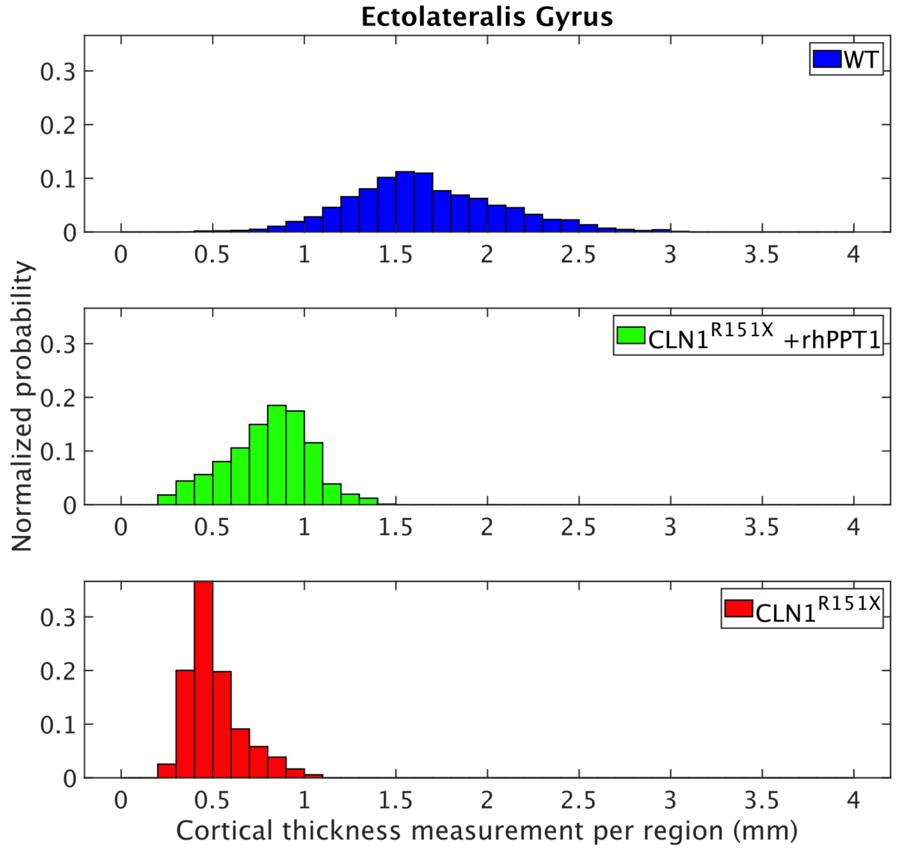

Entolateralis Gyrus

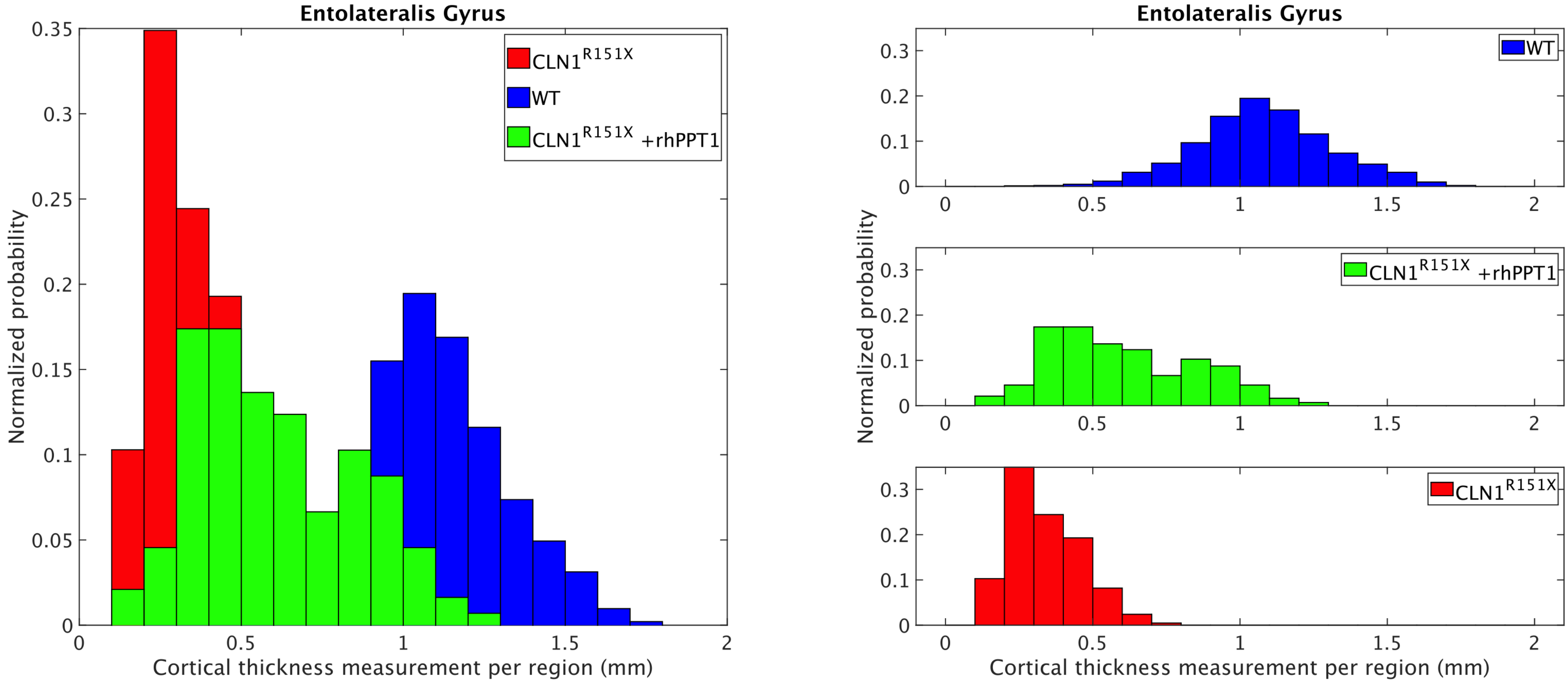

Lateral Gyrus

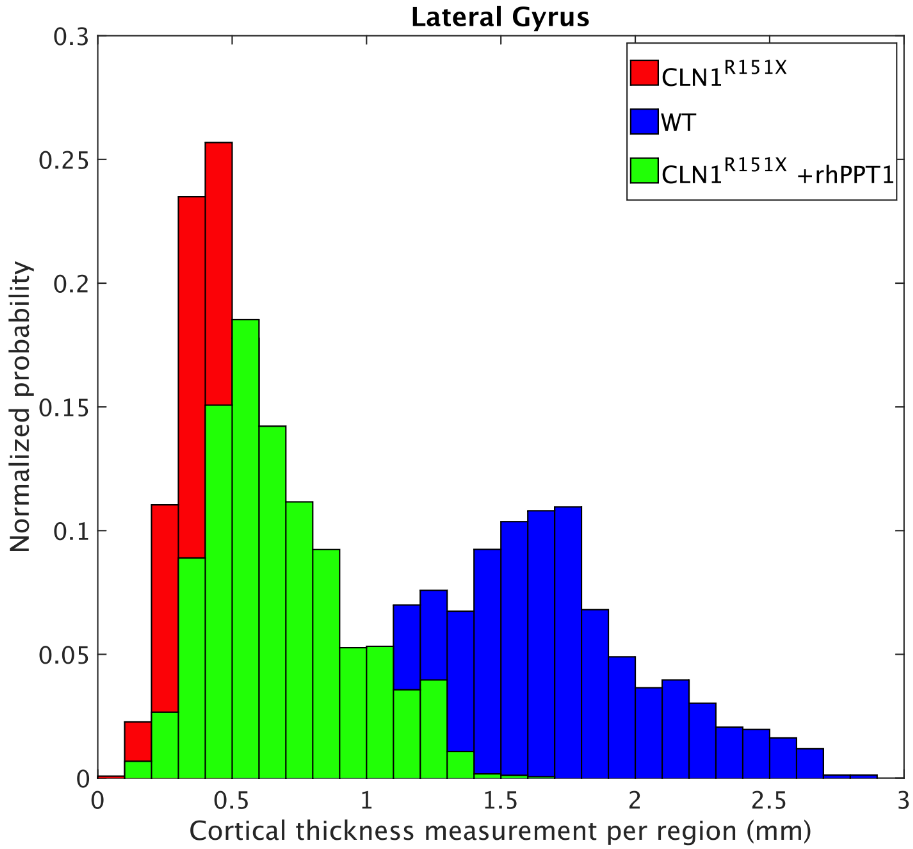

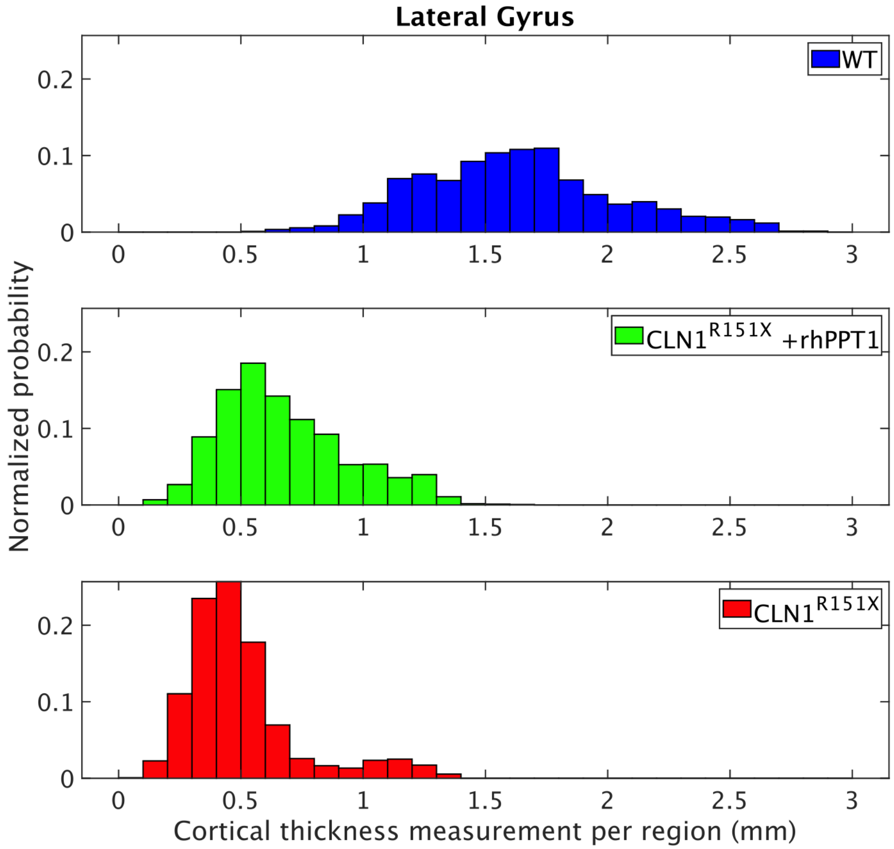

Occipital Lobe

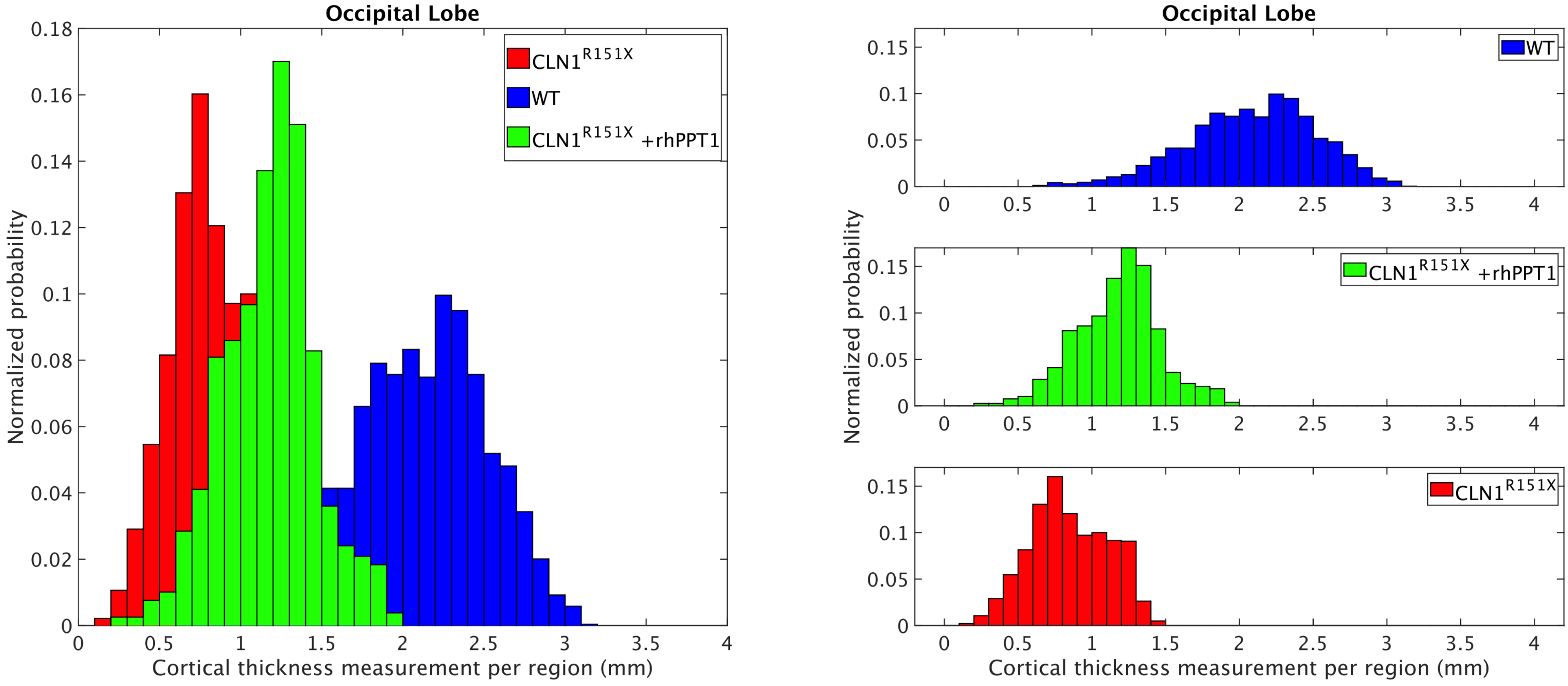

Orbital Gyrus

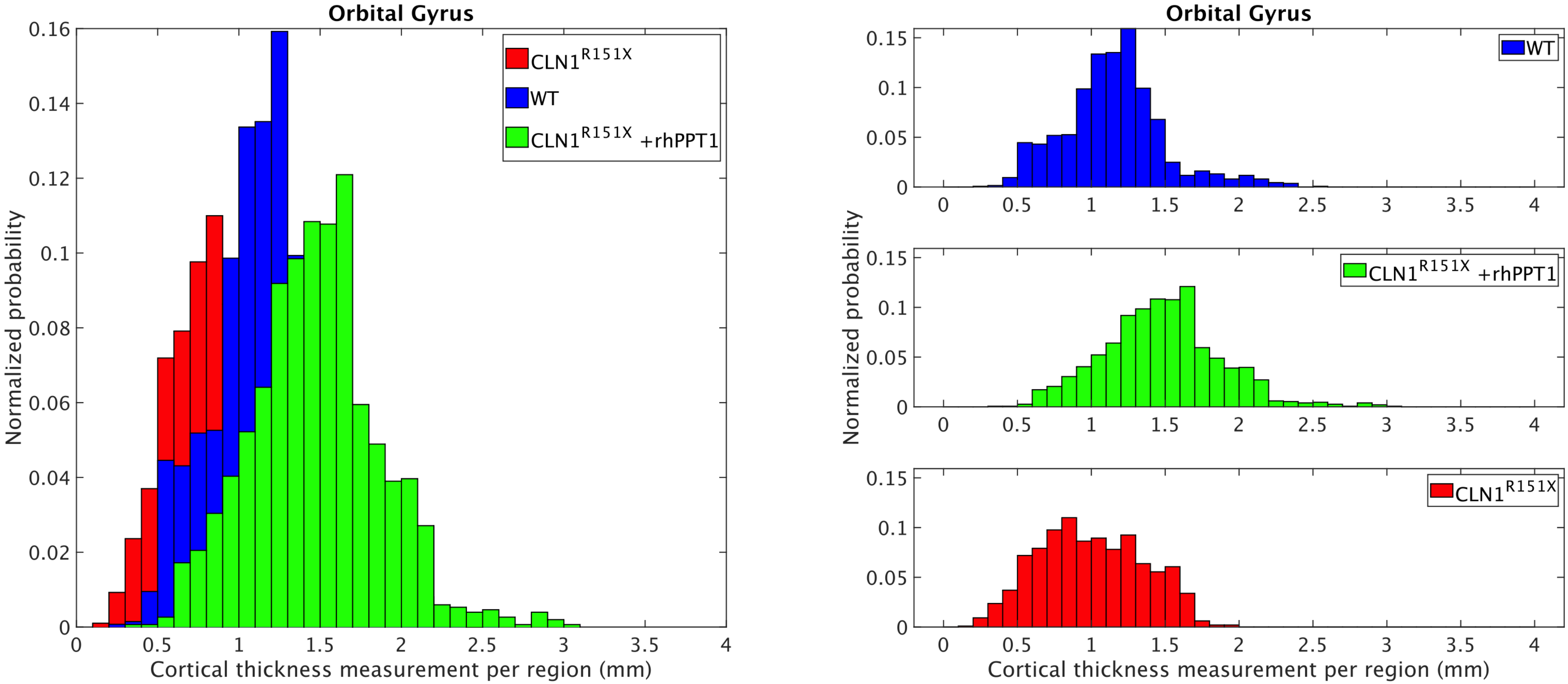

Orbitofrontal Gyrus

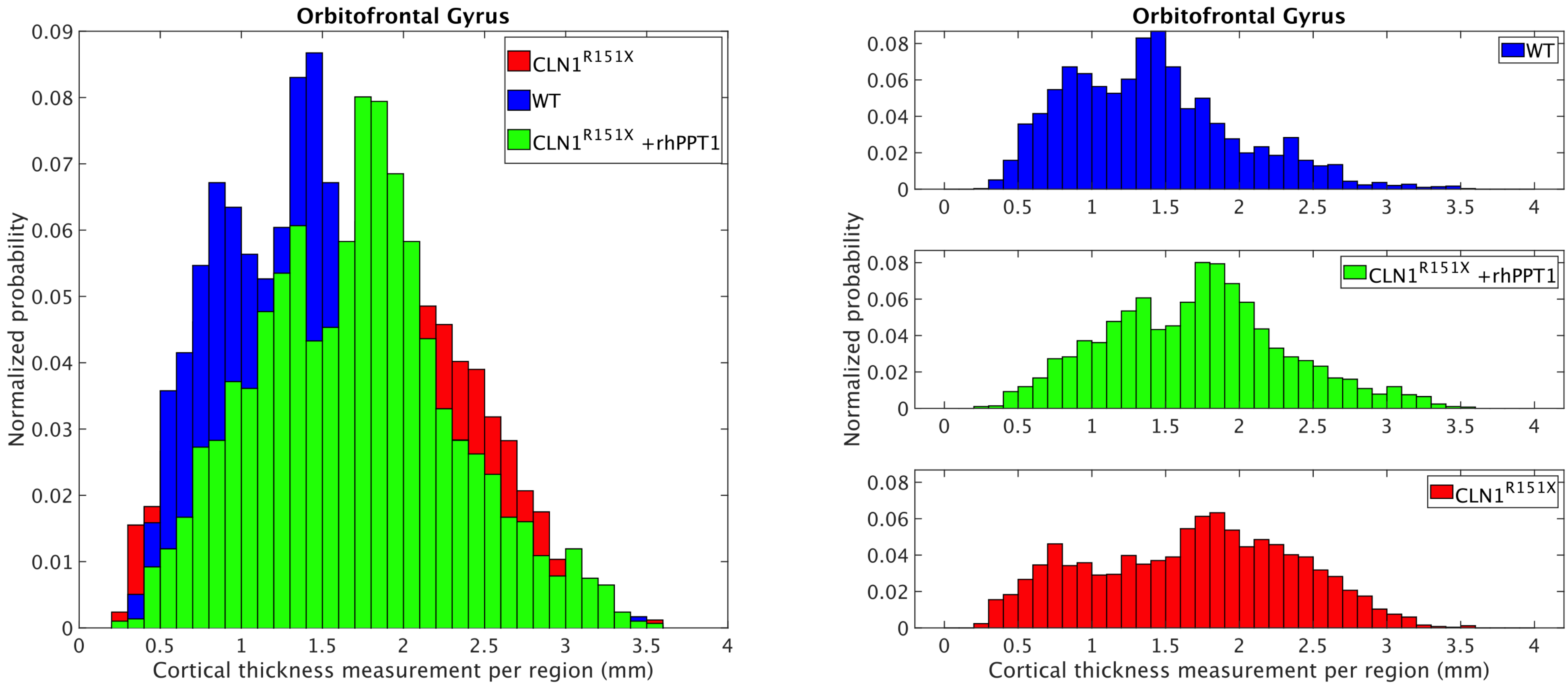

Parahippocampal Cortex

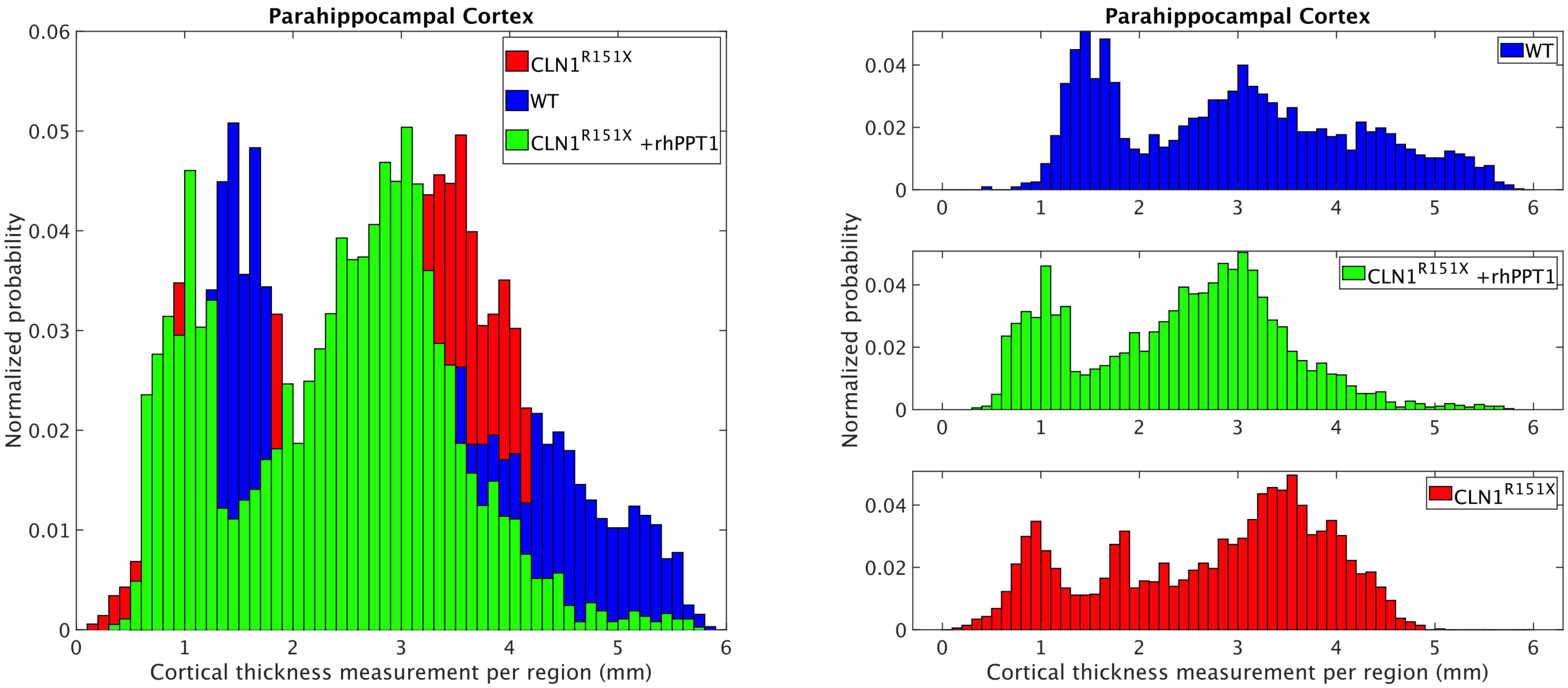

Posterior Sygmoideus Gyrus

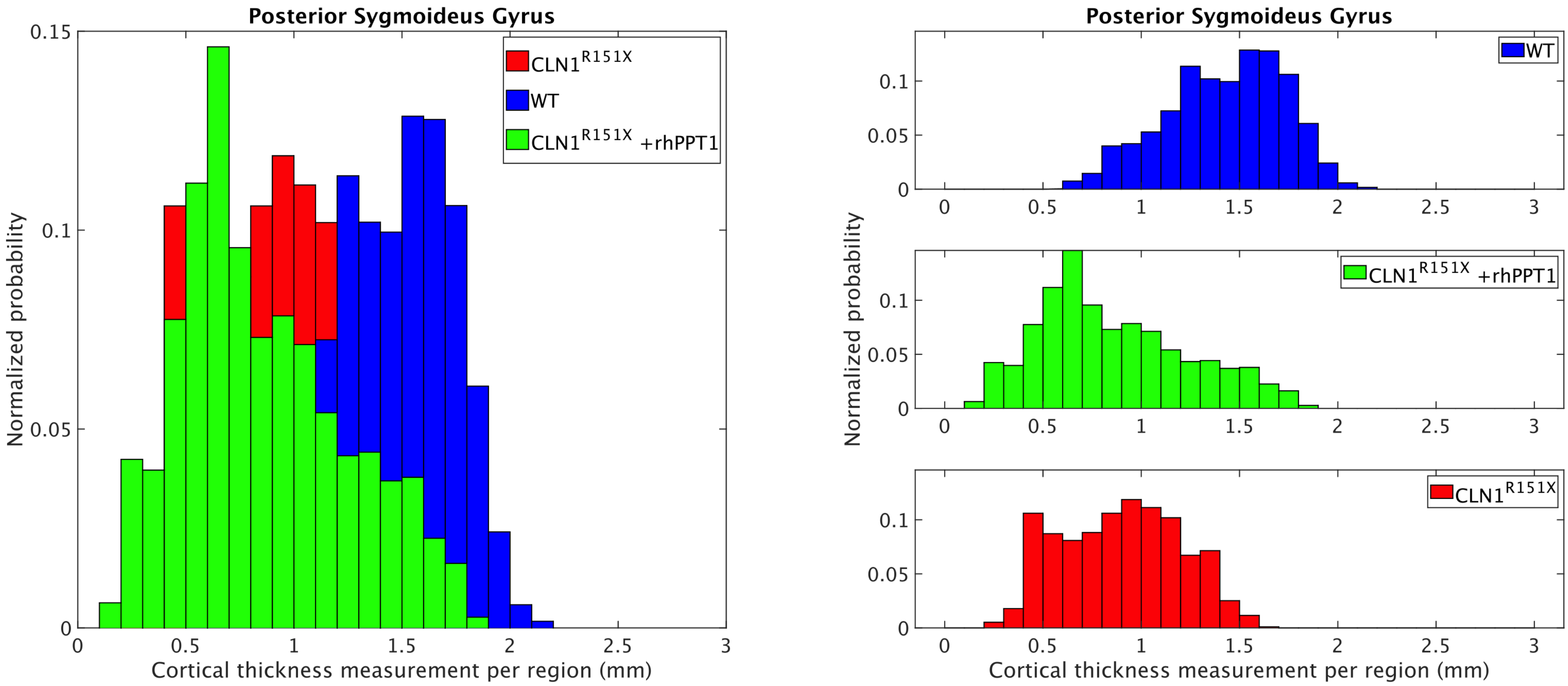

Posterior Sylvian Gyrus

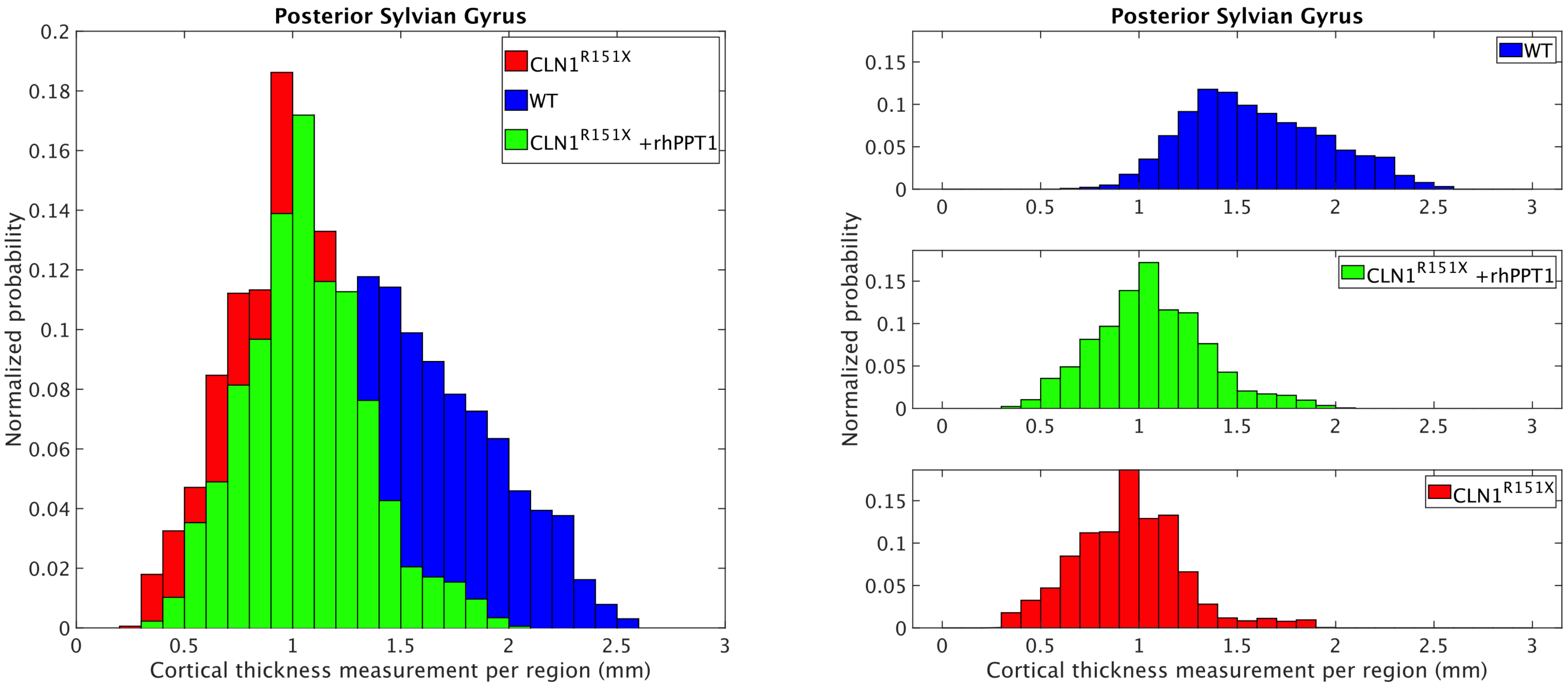

Gyrus Rectus

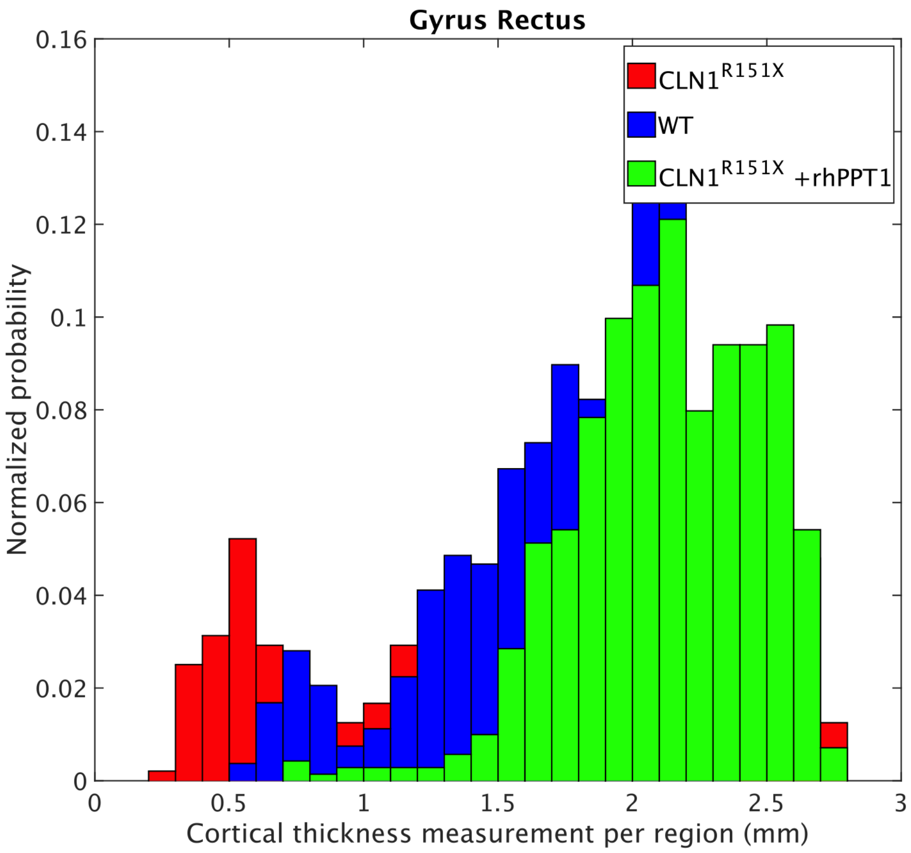

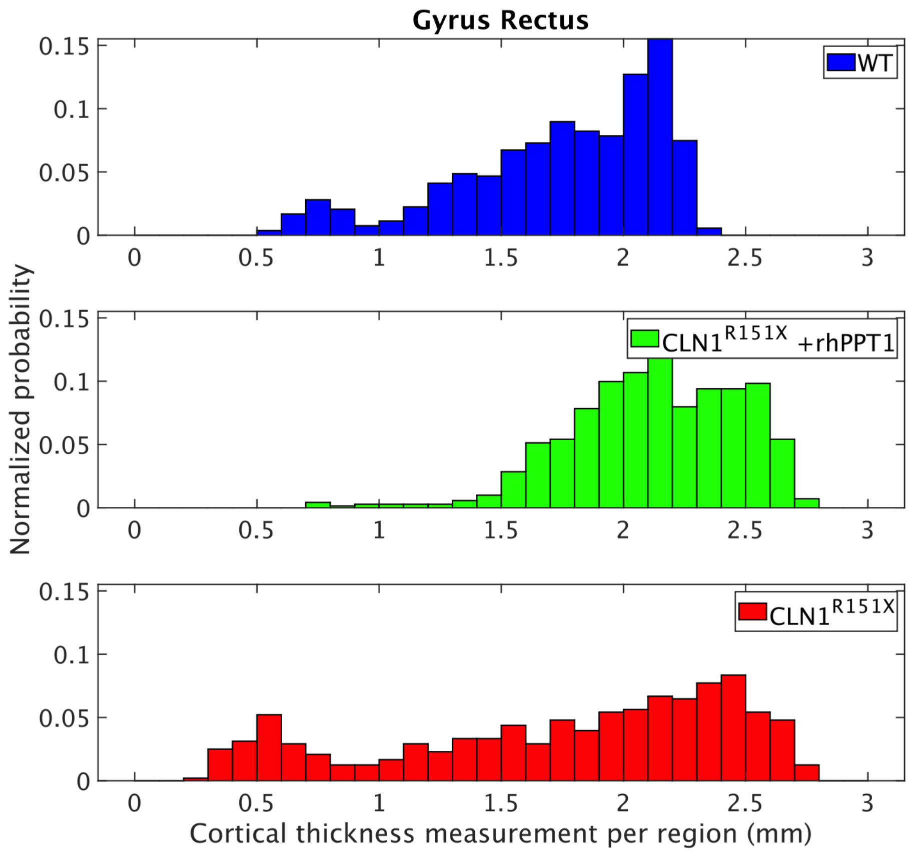

Suprasylvian Gyrus

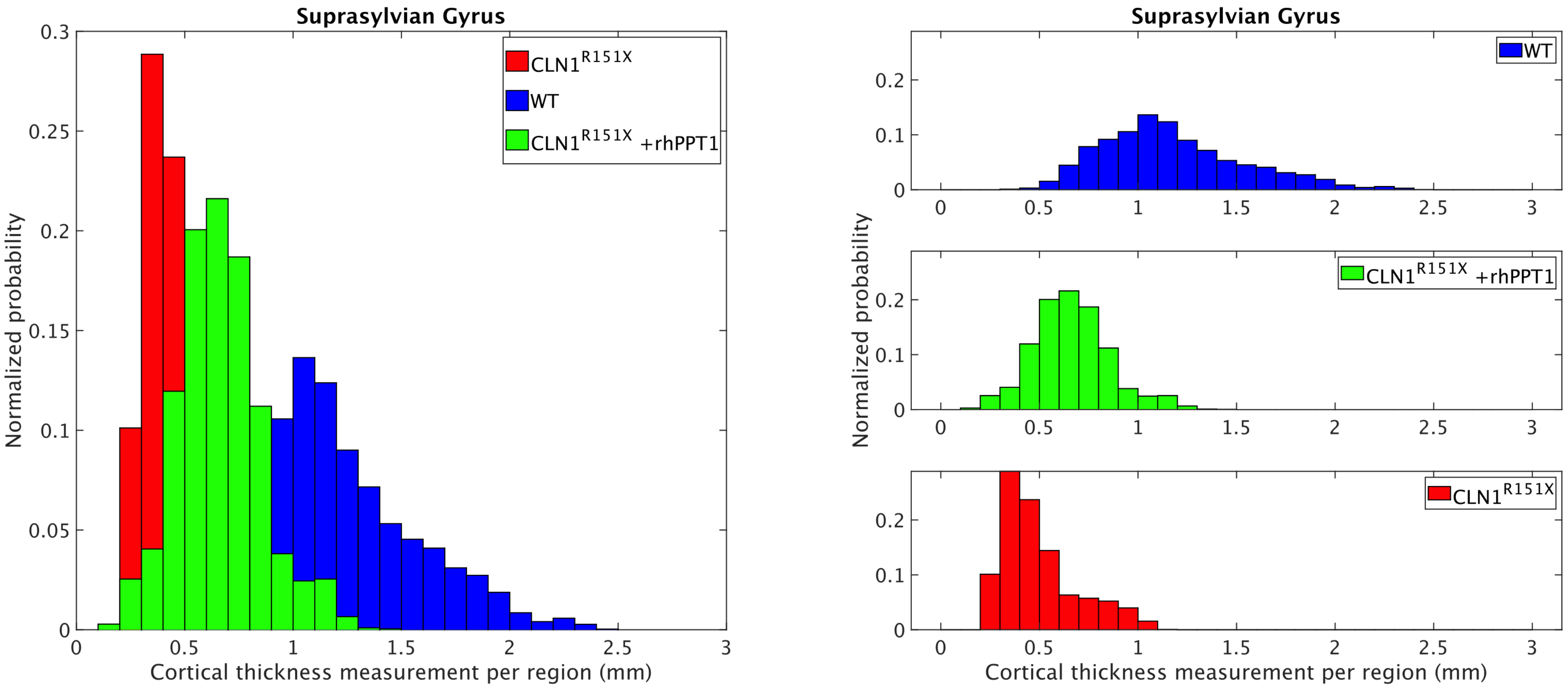

Sylvian Gyrus

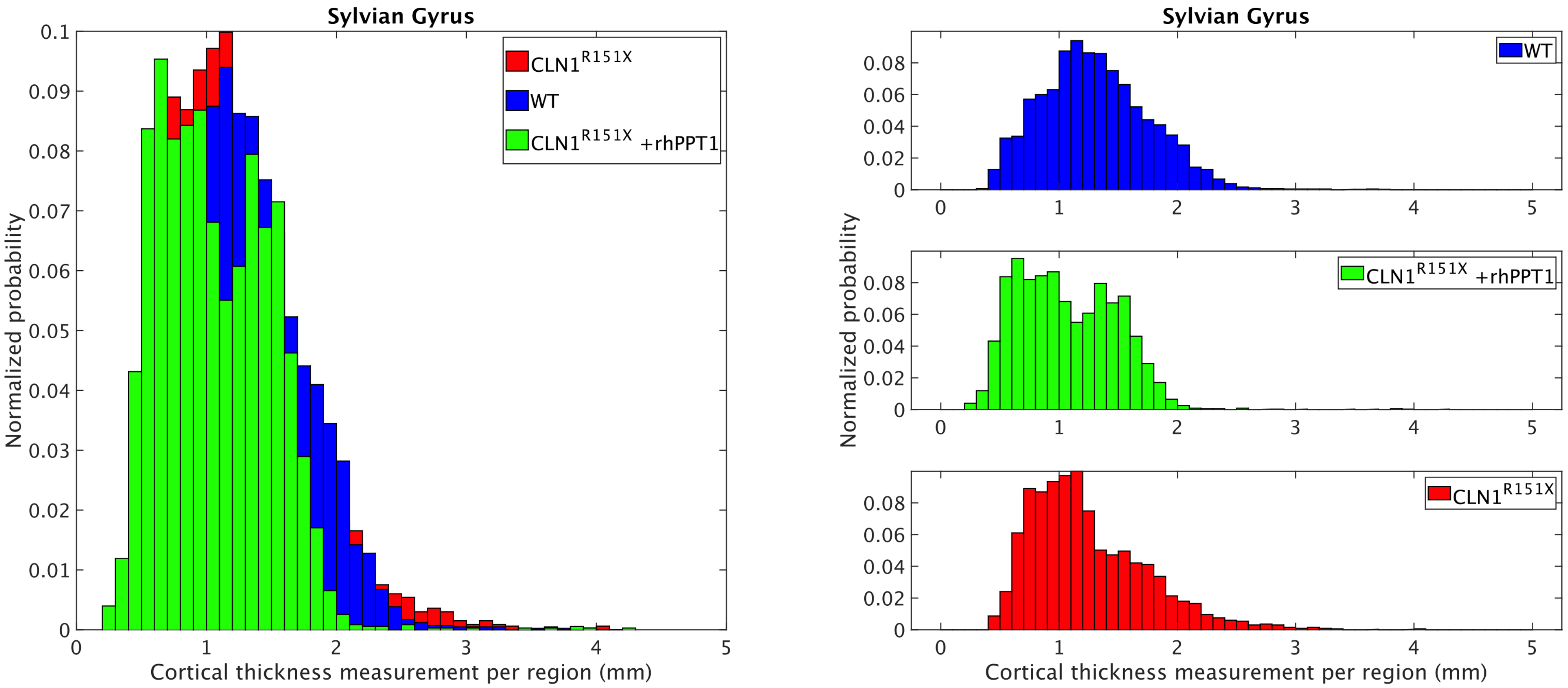

Temporal Lobe

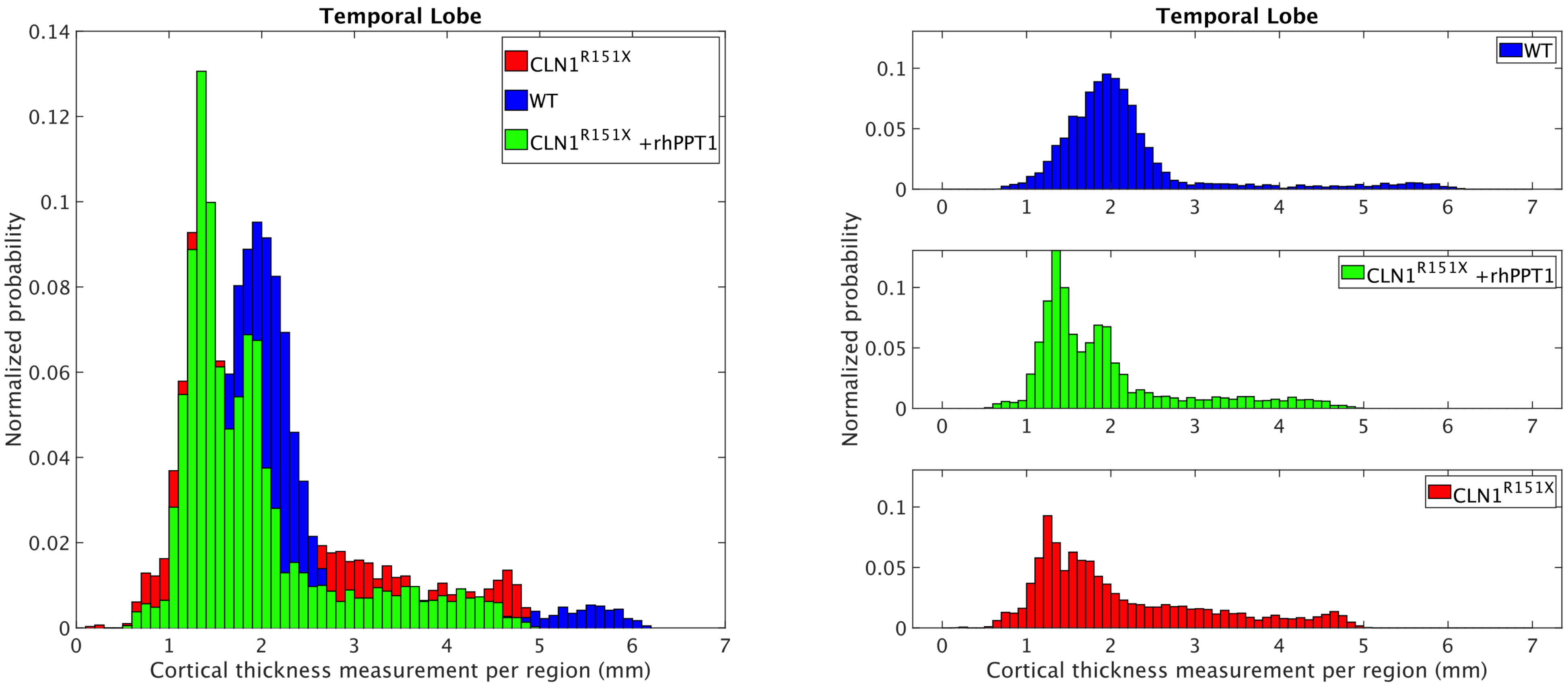

Supplement: Supplemental data set 3 [file jci-132-163107-s163.pdf]
